# Prefrontal Control over Motor Cortex Cycles at Beta Frequency during Movement Inhibition

**DOI:** 10.1016/j.cub.2014.10.043

**Published:** 2014-12-15

**Authors:** Silvia Picazio, Domenica Veniero, Viviana Ponzo, Carlo Caltagirone, Joachim Gross, Gregor Thut, Giacomo Koch

**Affiliations:** 1 Non-Invasive Brain Stimulation Unit, Clinical and Behavioral Neurology Department, IRCCS Santa Lucia Foundation, Rome 00179, Italy; 2 Centre for Cognitive Neuroimaging, Institute of Neuroscience and Psychology, University of Glasgow, Glasgow G12 8QB, UK; 3 Department of System Medicine, Tor Vergata University, Rome 00133, Italy; 4 Stroke Unit, Department of Neuroscience, Policlinic Tor Vergata, Rome 00133, Italy

## Abstract

A fully adapted behavior requires maximum efficiency to inhibit processes in the motor domain [1]. Although a number of cortical and subcortical brain regions have been implicated, converging evidence suggests that activation of right inferior frontal gyrus (r-IFG) and right presupplementary motor area (r-preSMA) is crucial for successful response inhibition [2, 3]. However, it is still unknown how these prefrontal areas convey the necessary signal to the primary motor cortex (M1), the cortical site where the final motor plan eventually has to be inhibited or executed. On the basis of the widely accepted view that brain oscillations are fundamental for communication between neuronal network elements [4–6], one would predict that the transmission of these inhibitory signals within the prefrontal-central networks (i.e., r-IFG/M1 and/or r-preSMA/M1) is realized in rapid, periodic bursts coinciding with oscillatory brain activity at a distinct frequency. However, the dynamics of corticocortical effective connectivity has never been directly tested on such timescales. By using double-coil transcranial magnetic stimulation (TMS) and electroencephalography (EEG) [7, 8], we assessed instantaneous prefrontal-to-motor cortex connectivity in a Go/NoGo paradigm as a function of delay from (Go/NoGo) cue onset. In NoGo trials only, the effects of a conditioning prefrontal TMS pulse on motor cortex excitability cycled at beta frequency, coinciding with a frontocentral beta signature in EEG. This establishes, for the first time, a tight link between effective cortical connectivity and related cortical oscillatory activity, leading to the conclusion that endogenous (top-down) inhibitory motor signals are transmitted in beta bursts in large-scale cortical networks for inhibitory motor control.

## Highlights

• r-IFG/l-M1 and r-preSMA/l-M1 connectivity increases during NoGo trials
• Motor inhibitory signals are transmitted cortically in beta bursts
• Effective connectivity is linked to beta oscillatory activity

## Teaser

Picazio et al. examine the prefrontal-to-motor cortex effective connectivity in a Go/NoGo task as a function of delay from (Go/NoGo) cue onset by using double-coil transcranial magnetic stimulation and electroencephalography. They reveal that in NoGo trials only, causal inhibitory influences from prefrontal to motor cortex cycle at beta frequency.

## Results

### Double-Coil TMS Experiment

**Figure 1:**
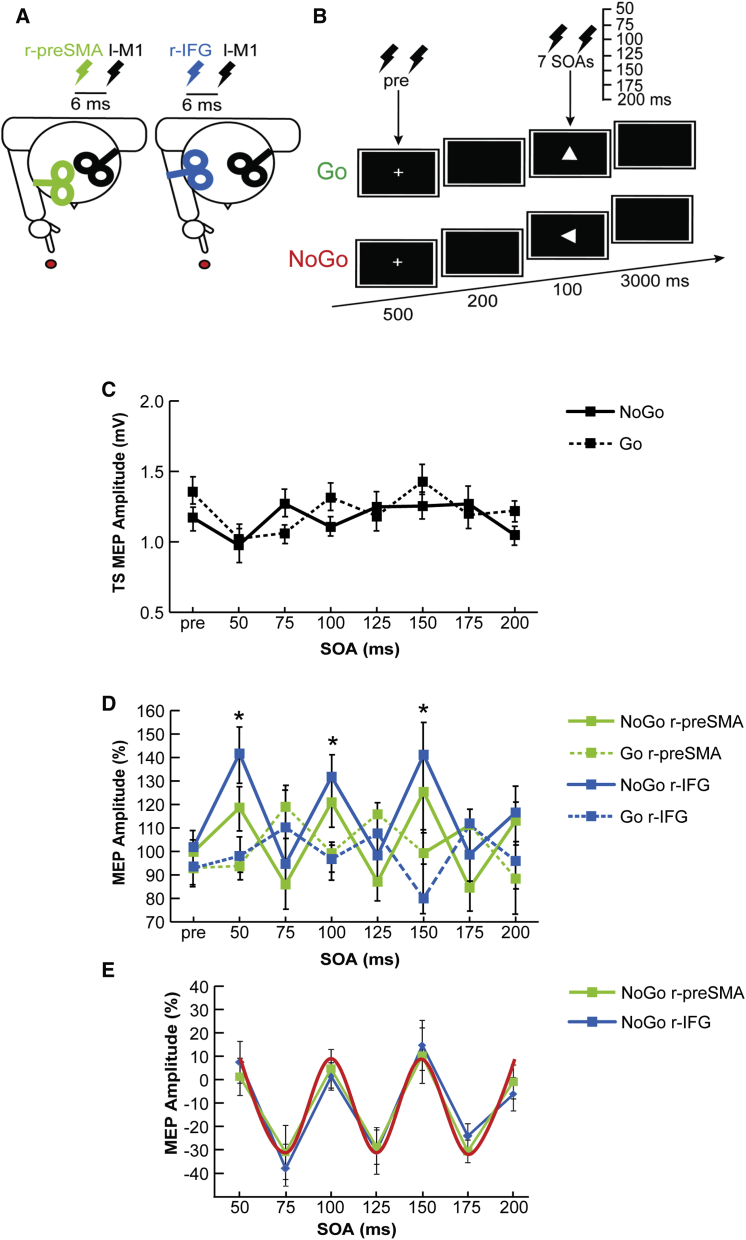
Experimental Setting and Results (A) Schematic drawing of coil positioning on the head. A TMS pulse was applied over l-M1 (TS) either alone or proceeded by a CS (CS + TS) delivered 6 ms earlier over the r-IFG/r-preSMA. (B) Single (TS) or paired pulse (CS + TS) TMSs were applied during the fixation cross presentation (pre) or during different delays (SOAs) after the presentation of the Go/NoGo cue during a premovement period (average RT was 428 ms). The task entailed the presentation of four types of white isosceles triangles pointing upward, downward, rightward, or leftward against a black background. Subjects were instructed to press a key with the right index finger whenever a white triangle pointed either up or down (Go stimulus) and to refrain from pressing the key whenever the triangle pointed toward the right or left (NoGo stimulus). (C) MEP amplitude (mV) for TS alone for each CS site (r-IFG/r-preSMA) at different SOAs for Go and NoGo trials. (D) MEP amplitude, expressed as percentage of change in comparison to TS alone, after r-IFG or r-preSMA conditioning at different SOAs for both Go and NoGo trials. (E) Group-averaged MEP amplitudes (linearly detrended) for the NoGo condition. The best-fitting 20 Hz model cosine is superimposed (red line). Asterisks indicate p < 0.01. Here and elsewhere, graph bars represent mean SEs.

The real-time activity of right inferior frontal gyrus/left primary motor cortex (r-IFG/l-M1) and right presupplementary motor area/left primary motor cortex (r-preSMA/l-M1) connections was tested in healthy volunteers by double-coil transcranial magnetic stimulation (TMS) during the early response period of a simple visually cued Go/NoGo task (Figures 1A and 1B). To this end, we administered a conditioning stimulus (CS) over one of these right prefrontal areas followed by a test stimulus (TS) over left M1, always using a fixed CS-TS interstimulus interval of 6 ms (previously shown to probe instantaneous prefrontal-to-motor connectivity) [9, 10] (Figure 1A), but varying the delays of paired CS-TS TMS administration from the onset of the imperative (Go/NoGo) movement cue (stimulus onset asynchrony [SOA]: 50–200 ms, in steps of 25 ms) to probe for fluctuations of effective connectivity in these prefrontal/M1 networks over time (Figure 1B) during the execution of a Go/NoGo task [11]. We used motor-evoked potentials (MEPs) recorded from the first dorsal interosseus muscle of the right hand as dependent measure to verify the causal influence of conditioning pulses applied over either r-IFG or r-preSMA (for more information, see Supplemental Experimental Procedures available online). The experimental procedures were approved by the local Ethics Committee according to the Declaration of Helsinki.

Go/NoGo performance revealed a high accuracy rate in Go trials (99.3% ± 1.1%) and a low false alarm rate in NoGo trials (2.5% ± 2.1%), indicating good compliance of the participants. Reaction times (Go trials) were on average 428 ms (range across SOAs: 381–476 ms), indicating that all tested SOAs (50–200 ms) fell within an early premovement epoch corresponding to an early phase of motor planning.

In terms of cortical changes over time after Go/NoGo cueing, we first verified the time course of cortical excitability of l-M1 alone during the premovement period by analyzing MEP amplitude evoked by the TS without any CS. A main effect of SOA (F_7,63_ = 2.67; p = 0.018), but no significant modulation depending on the Go/NoGo condition or any interaction, was found for TS-induced MEP amplitude (Figure 1C). MEPs were reduced at 50 ms in comparison with all other SOAs, with the exception of the 200 ms SOA (all p < 0.05), indicating an unspecific decrease in l-M1 excitability immediately after the onset of the visual cue.

Testing r-IFG/l-M1 and r-preSMA/l-M1 effective connectivity by double-coil TMS (r-prefrontal CS/l-M1 TS) revealed a task-related temporal profile (Figures 1D and 1E), characterized by clear reverberant peaks of prefrontally enhanced l-M1 excitability at specific time points after the NoGo signal and a weaker (but counterphase) variation after the Go signal. Main effects of condition (Go versus NoGo) (F_1,9_ = 9.66; p = 0.012) and SOA (F_7,63_ = 2.30; p = 0.037) with a significant condition × SOA interaction (F_7,63_ = 7.71; p < 0.001) emerged on conditioned MEP amplitudes (Figure 1D). No effect of site (r-IFG versus r-preSMA) was found. MEPs were markedly and selectively increased for NoGo trials at 50 ms (p = 0.003), 100 ms (p = 0.002), and 150 ms (p = 0.001) after the stimulus onset in comparison to Go trials (Figure 1D). Importantly, when comparing these intervals to the conditioned MEP amplitude collected during the precue period, we found a significant difference at all these peaks in the NoGo condition (precue versus 50 ms: p = 0.028; precue versus 100 ms: p = 0.027; precue versus 150 ms: p = 0.029). This was not the case for the Go condition.

We then performed a curve-fitting procedure to examine whether the recurrently enhanced l-M1 excitability in the NoGo condition, peaking every 50 ms, can be accounted for by a cyclic pattern. Permutation tests revealed that cosine models in the beta-frequency range (from 17–23 Hz) significantly fitted the MEP data when the CS was delivered over r-IFG (20 Hz cosine being the best-fitting model; Figure 1E). The same results were found when the CS was applied over r-preSMA (best fit: 20 Hz cosine; Figure 1E). This supports the existence of a beta-oscillation underling group-averaged MEP amplitude modulation during NoGo trials, as tested by prefrontal conditioning. Crucially, cosine fitting in the beta range was also significant at the individual level (as confirmed by single-subject analysis of the MEP measure of effective connectivity) for NoGo data, but not for Go data (see Supplemental Results and Figures S1 and S2).

**Figure 2:**
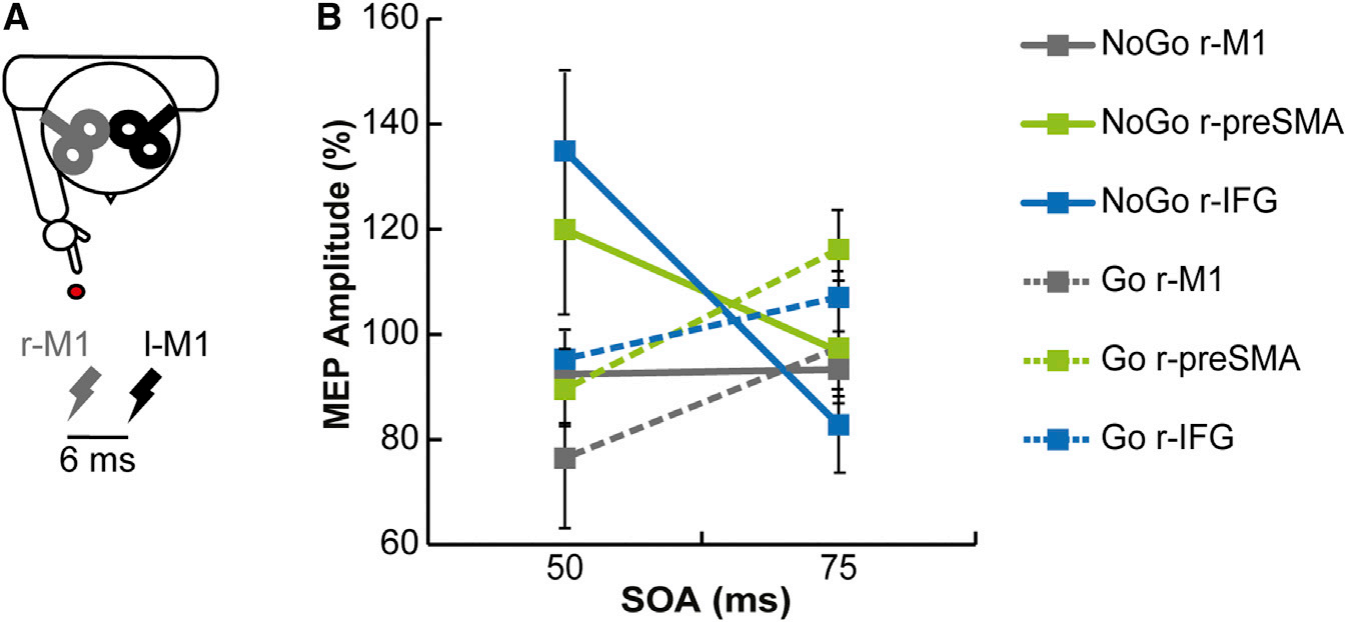
Experimental Setting and Results of the Control Experiment (A) Schematic drawing of coil positioning on the head. TMS was applied either alone over l-M1 or proceeded by a CS delivered 6 ms earlier over the r-M1, r-IFG, or r-preSMA at two different delays (50 ms and 75 ms) after the onset of the Go/NoGo cue. (B) MEP amplitude, expressed as percentage of change in comparison to TS alone, after r-M1, r-IFG, or r-preSMA conditioning at different SOAs for both Go and NoGo trials.

We conducted a control experiment exploring r-M1/l-M1 connectivity (Figure 2A) to ensure that the results obtained when the CS was applied over the r-IFG or r-preSMA were due to the conditioning effect of the pulse over the targeted prefrontal areas and not due to a spread of activation to nearby r-M1. We chose two SOAs (50 ms versus 75 ms) showing significant variations in inferred connectivity in the main experiment (i.e., r-IFG/l-M1 and r-preSMA/l-M1). r-M1/l-M1 connectivity did not show a similar modulation of corticospinal excitability as r-IFG/l-M1 and r-preSMA/l-M1 probes did (main effect of site: F_2,14_ = 6.79; p = 0.008) (Figure 2B).

### TMS-EEG Experiment

**Figure 3:**
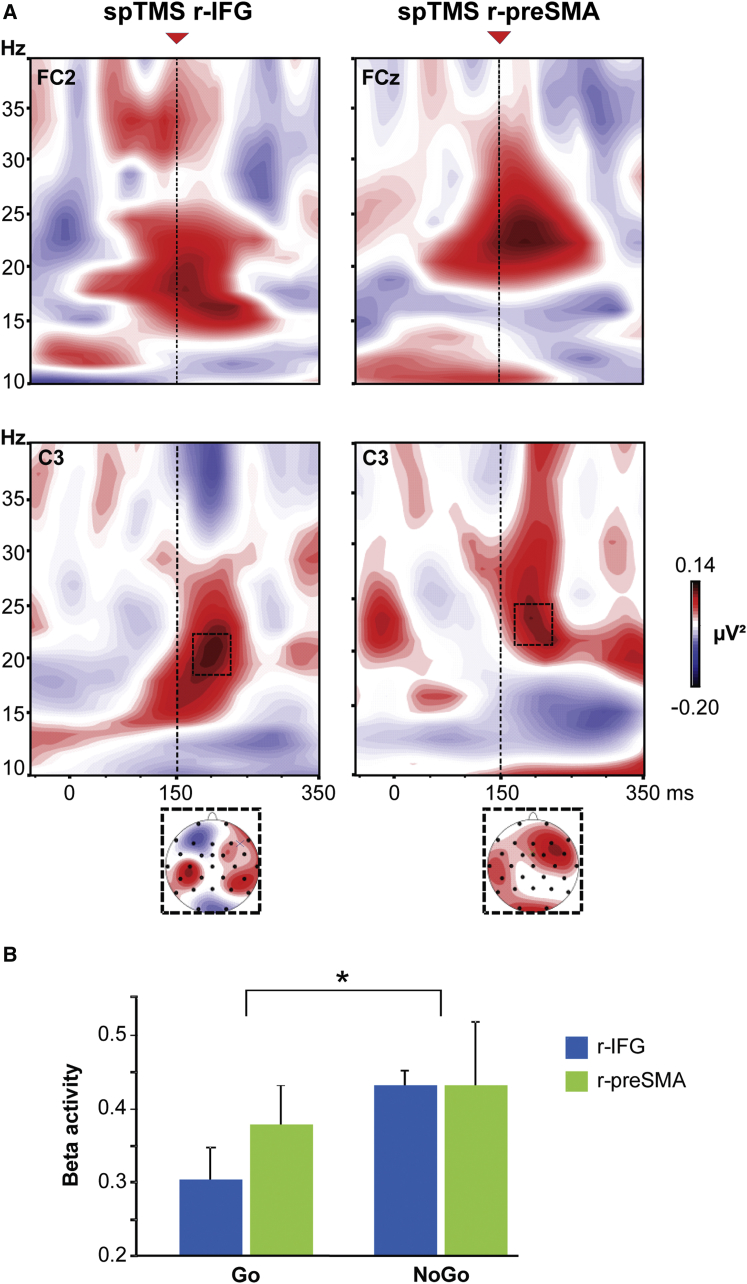
TMS-EEG Experiment Results (A) Time-frequency plots for each TMS site (r-IFG on the left, r-preSMA on the right) are shown for C3 and for two electrodes close to the conditioning site. The activity recorded for the Go condition was subtracted from the activity recorded during the NoGo condition. Zero represents time of visual cue onset, whereas the red arrows indicate the timing of pulse delivery (150 ms after cue onset). The dotted squares indicate a significant difference between conditions and the corresponding topographical map. (B) Beta power plotted as function of condition and TMS site. Asterisk indicates p < 0.05.

We conducted a TMS-electroencephalography (EEG) experiment to directly probe instantaneous oscillatory network activity through the administration of a single TMS pulse over r-IFG or r-preSMA at 150 ms into the execution of the Go/NoGo paradigm while simultaneously recording EEG [8, 12, 13]. This 150 ms SOA was selected on the basis of the findings obtained in the double-coil TMS experiment. Figure 3A shows a time-frequency plot after subtraction of the evoked activity recorded in the NoGo condition minus Go condition for the two electrodes close to the (conditioning) TMS sites (FC2 for r-IFC and FCz for r-preSMA) and for the electrode closest to l-M1 (C3). This demonstrates dominant sustained beta increases (20–23 Hz peak frequency, 50–250 ms after cue onset), starting at early time points over prefrontal sites and spreading to left central sites after both r-IFG and r-preSMA stimulation in NoGo as compared to Go trials (Figure 3A). In line with the MEP results, the EEG topography (after 150 ms) revealed a right frontal-left central signature (Figure 3A, map inset). A main effect of condition (Go versus NoGo) (F_1,5_ = 15.8; p = 0.01) was found for C3 beta power, with higher beta power during the NoGo condition than the Go condition (Figure 3B). No effect of site was found on C3 beta activity, further suggesting that r-IFG and r-preSMA activation can result in an M1 excitability modulation by acting on the same oscillatory activity. This therefore links the cyclic pattern of prefrontal-M1 connectivity in the beta range as revealed by double-coil TMS to oscillatory brain activity as recorded by EEG (NoGo > Go), without being able to resolve where the NoGo-related surface EEG activity at beta frequency is generated (i.e., whether it is predominantly restricted to M1 or prefrontal areas, or whether it is reflecting network activity).

**Figure 4:**
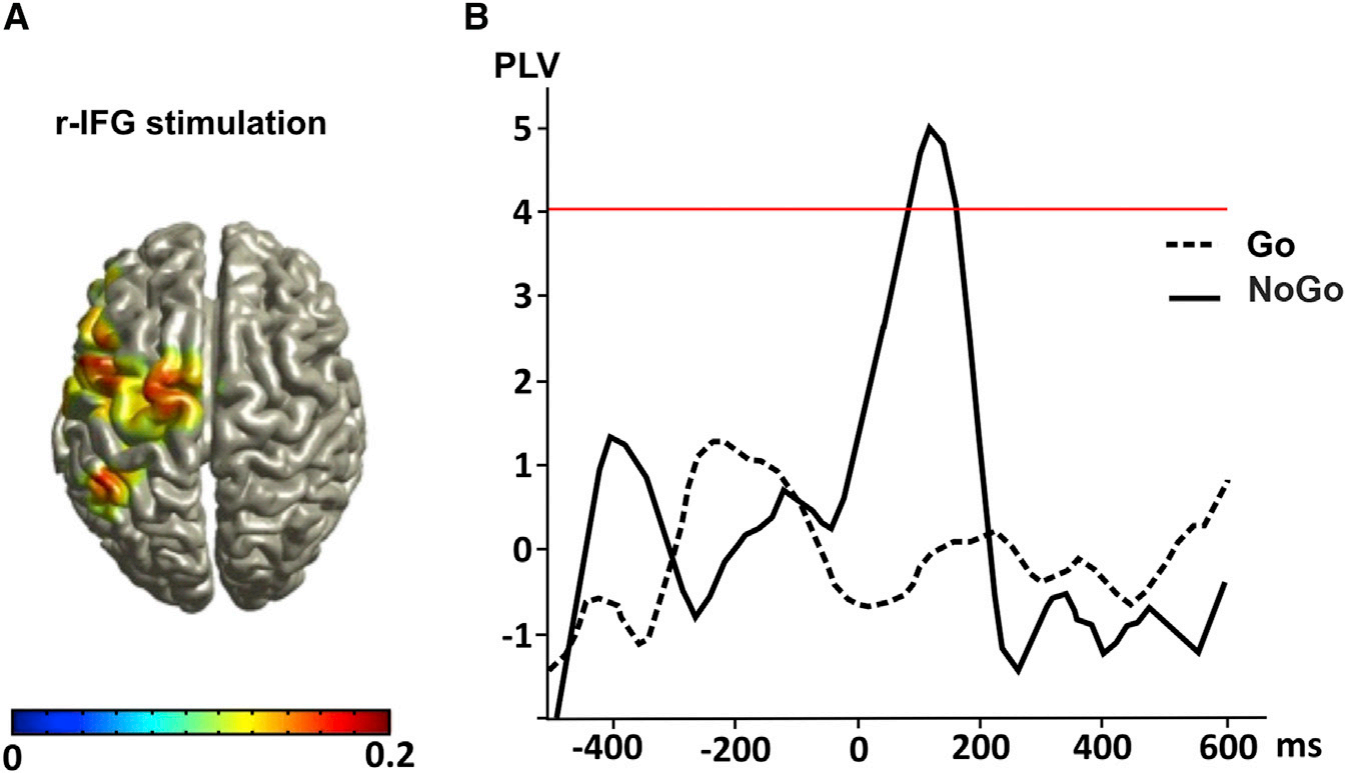
Source Analysis and PLV (A) Estimate of most prominent beta sources calculated for a 100 ms time window after the TMS pulse on the relative changes between the NoGo and Go condition. (B) Phase-locking time series, plotted in units of SD, of the baseline for the Go and NoGo condition. The horizontal red line indicates the 99% confidence level.

To address this point, we performed additional analyses in source space (see Supplemental Experimental Procedures). Given the restrictions of the EEG recording (i.e., limited number of electrodes and its low spatial resolution), we limited the source analysis to the IFG condition (with M1 and preSMA not being separable because of their proximity). The source analysis that was performed to identify the generator of beta activity evoked by TMS over r-IFG during the NoGo trials revealed left M1 as a prominent source (Figure 4A). To investigate functional connectivity between this area and r-IFG, we calculated phase-locking value in the beta band for Go and NoGo trials in the r-IFG condition (see Supplemental Experimental Procedures). As shown in Figure 4B, a significant phase coherence increase between the two areas was present for the NoGo, but not the Go, condition, and this occurred during the relevant time interval (around 50–150 ms). Hence, cyclic patterns in prefrontal-M1 connectivity in the beta range (NoGo > Go), as inferred from double-coil TMS, not only coincide with EEG beta increases over areas of this network but also can be linked to more direct EEG measures of functional network activity in this frequency band.

Finally, we calculated the phase-locking index (PLI) across trials for the electrode closest to M1 (C3) to test whether the activation of r-IFG realigns the instantaneous beta phase over the motor cortex. This analysis revealed that the NoGo condition was characterized by a significantly increased intertrial phase consistency in the beta range when compared to the Go condition (see Supplemental Results and Figure S4).

## Discussion

The current findings provide first-time evidence linking reverberations in effective strength of cortical connectivity with underlying cortical oscillations in the motor system. The early causal inhibitory influences of prefrontal cortex on M1 activity are not transferred in a discrete manner but follow an oscillatory beta rhythm. Notably, this coincides with the presence of beta oscillations in the prefrontal-motor pathway, as revealed by TMS-EEG experiments. These results imply that the transmission of causal influences in cortical networks is related to channels of communication tuned to specific frequencies, here in the beta rhythm. The findings provide important information on the implication of oscillations in brain function in general, namely on the open question of to what extent cortical rhythms underlie network communication per se. Specifically, they also provide novel information on the causal implication of beta rhythms in cortical inhibitory motor control.

There is accumulating evidence for a causal implication of brain oscillations [14]. Several recent behavioral studies [15–17] unveiled rhythmic fluctuations in performance measures after temporal reset of visual or attentional processing due to the presentation of a discrete sensory event (see [18] for a review). This coincided with the periodicity of concurrently recorded rhythms of the visual brain, revealed by simultaneous EEGs [17]. Similar to the above studies [15–17], our data set examines cycles in behavioral measures as a function of delay from an external event (here, the imperative Go/NoGo signal). Here, we tested the instantaneous communication between two brain areas of a network at different time points, unlike the above studies, which tested visual performance (and hence excitability) at a single time point. By showing that this measure of instantaneous effective connectivity cycles at beta frequency and coincides with beta oscillation, we demonstrate in novel ways that cortical connectivity per se relates to rhythms of the brain. Importantly, no such cycles were observed with single-pulse TMS over motor cortex following the presentation of the NoGo cue, ruling out an explanation of fluctuating motor cortex excitability in our case. In addition, we did not find motor cortex excitability as tested by single-pulse TMS to inversely relate to beta power, i.e., excitability was not globally reduced in the NoGo condition for which higher beta power was observed (Figure 1C). Instead, when the CS was applied over prefrontal areas, MEP amplitudes were enhanced at 50 ms, 100 ms, and 150 ms after the NoGo cue but showed a trend of being reduced at 75 ms, 125 ms, and 175 ms after the NoGo cue (Figure 1D). Therefore, despite the fact that the required final output was inhibiting the action, we found an alternating pattern of increasing and decreasing MEP amplitudes at specific time intervals in an early phase of motor planning (0–200 ms after Go/NoGo cueing). This points to beta phase playing a functional role in this interval rather than beta amplitude, and it implies underlying changes in primary motor cortex connectivity rather than in excitability, as further confirmed by the phase-locking results.

Our results therefore provide direct support for the suggestion that brain oscillations are related to communications within neural networks [4–6]. In particular, they are in accord with the views that synchronization of oscillatory activity reflects spectral fingerprints of large-scale neuronal interactions [5, 19] linking beta oscillations with a large-scale inhibitory motor control network [20, 21] and that communication in these networks occurs in bursts at their typical frequencies, as predicted by the communication-through-coherence theory [6, 22]. The current results are also in line with the findings that beta oscillations seem prominently involved in top-down (feedback) interactions within cortical networks, as compared to higher frequency oscillations in the gamma band [5, 23, 24], and with a recent TMS-EEG study showing that the inferior prefrontal cortex has specific resonant properties in the beta-frequency range [25]. Here, we show for the first time that spectral EEG fingerprints of top-down inhibitory control signals are also visible in direct measures of corticocortical interactions of the corresponding network (tested by double-coil TMS). The main interpretation of our results is therefore that functional coupling of the prefrontal areas with the motor cortex in NoGo conditions occurs at a beta rhythm. We excluded that the NoGo cue induced similar oscillation in local l-M1 excitability independently from prefrontal input because in the control experiment, r-M1 pulses did not elicit rhythmicity in l-M1 MEPs under otherwise identical conditions. However, we cannot infer based on our data how this communication comes about. It is possible that beta oscillations, which are prominently represented in both areas, become phase aligned, opening the communication between these areas. Another possibility is that the NoGo cues directly induce beta oscillation in the prefrontal areas and that the periodicity in the connected motor cortex is a natural consequence of this prefrontal oscillation (as TMS pulses in connected M1 may be more potent when applied in coincidence with up states of excitability in right frontal cortex). Nonetheless, both scenarios go along with prefrontal areas showing cyclic changes in strength of coupling (i.e., effective connectivity) at beta frequency.

In terms of the anatomy of inhibitory motor control, the interactions of both the r-IFG and the r-preSMA with the left M1 revealed a similar temporal profile during NoGo trials. This result is in accordance with increasing evidence indicating that both areas are crucial for response inhibition, albeit with a possible lateralization to the right hemisphere, suggesting that these two regions work together to exert a causal control in the early phases of movement inhibition [2, 3]. Although no clear difference between r-IFG and r-preSMA emerged, it has to be noted that the beta-power increase in the NoGo condition, which was found during the TMS-EEG experiment, was predominantly due to the modulation caused by the r-IFG TMS (Figure 3B). Another limitation of the present study is the sampling rate used to acquire the MEP data (40 Hz). As a consequence, we cannot exclude that frequencies higher than beta are involved in the coupling between the prefrontal areas and the primary motor cortex or that the results found are due to aliasing of higher frequencies, although the EEG results covering a bigger frequency range do point to the same frequencies, i.e., beta frequency.

In terms of rhythms of the motor system, our TMS-EEG data link the findings of reverberant connectivity at beta frequency to central beta-band activity that has traditionally been viewed to reflect an idling state of motor cortices [26], supported by the findings that beta activity is attenuated during voluntary movements. However, accumulating evidence indicates that beta-band activity is causally involved in motor control [27, 28] and actively engaged in promoting the existing motor state and preventing new movement initiation [29]. In addition to inhibitory control of action by beta oscillation, recent evidence also points toward inhibitory control by means of alpha oscillations [21, 30]. These findings are not mutually exclusive because here, we focus on the causal influence of prefrontal areas on motor cortex excitability (i.e., corticocortical connectivity), whereas the former studies focused on upper alpha activity at sensorimotor cortices and corticospinal activity per se [30]. Taken together, our data reveal for the first time that beta activity reflects bursts of top-down, corticocortical network interactions serving a role in premovement inhibition of motor acts. This is more in line with the hypothesis that beta oscillations may predominantly reflect endogenously driven processes serving the maintenance of the status quo of a current sensory-motor or cognitive state [31] than the initial idling-state hypothesis.

### Conclusions

Our work provides novel evidence for a link between effective cortical connectivity and related cortical oscillatory activity in mediating response inhibition that has not been observed before. These findings could have important implications in unraveling abnormalities of motor and cognitive inhibition in several pathological conditions, including Parkinson’s disease, autism, and schizophrenia [32, 33], and could be important in building novel therapeutic strategies based on invasive or noninvasive cortical stimulation or neuroprosthesis.

## Author Contributions

S.P. and G.K. conceived the study. S.P. and D.V. performed the experiments and data analysis. J.G. performed data analysis. V.P. performed the experiments. C.C., G.T., and G.K. supervised the experiments. S.P., D.V., J.G., C.C., G.T., and G.K. wrote the paper.

## Supplemental Information

Document S1. Supplemental Results, Supplemental Experimental Procedures, and Figures S1–S4

Document S2. Article plus Supplemental Information
