# Supplementary material for: Prefrontal Control over Motor Cortex Cycles at Beta Frequency during Movement Inhibition
Source: Curr Biol. 2014 Dec 15;24(24):2940–5. doi: 10.1016/j.cub.2014.10.043 (PMC4274313; doi:10.1016/j.cub.2014.10.043)
Supplement: Document S1. Supplemental Results, Supplemental Experimental Procedures, and Figures S1–S4 [file mmc1.pdf]

Current Biology, Volume *24*

Supplemental Information

Prefrontal Control over Motor Cortex Cycles at Beta Frequency during Movement Inhibition

Silvia Picazio, Domenica Veniero, Viviana Ponzo, Carlo Caltagirone, Joachim Gross, Gregor Thut, and Giacomo Koch

Supplemental Figure and Legends

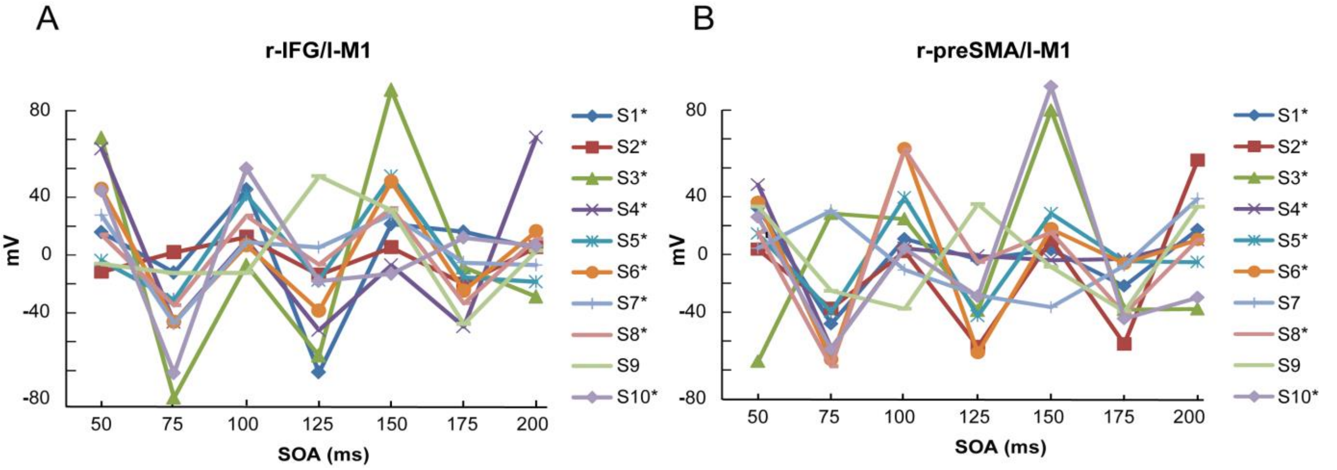

Figure S1 Related to Figure 1. Single-subject MEP-amplitudes data (linearly detrended) for the Nogo trials for the two conditioning sites, r-IFG (A) and r-preSMA (B). Asterisks indicates a significant beta band fitting (*p*<0.05).

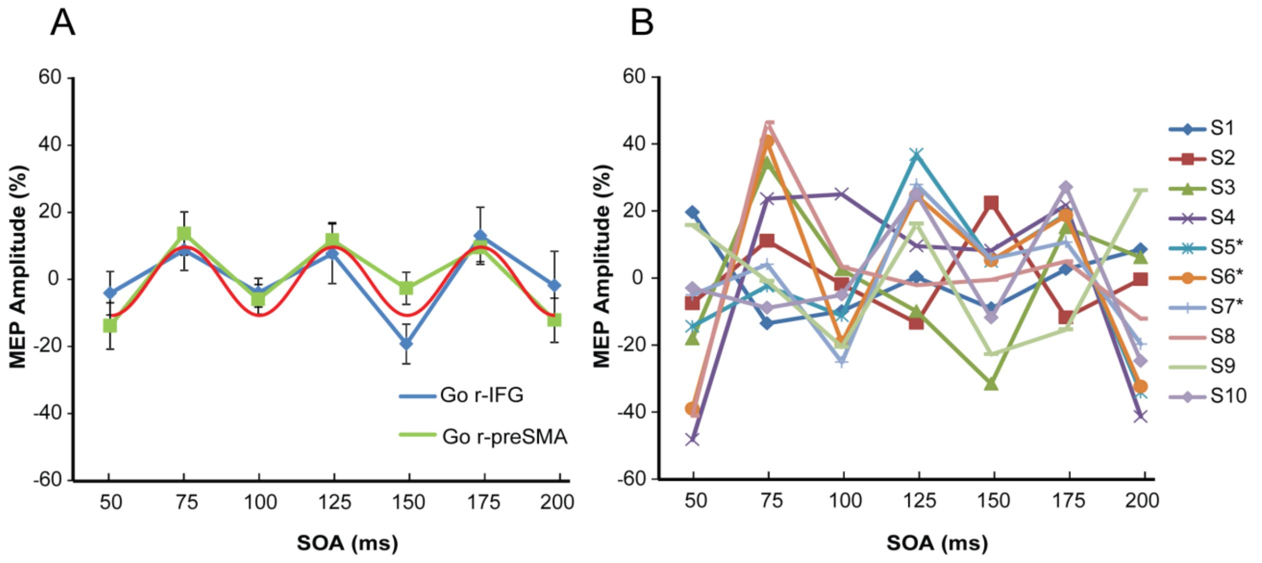

Figure S2 Related to Figure 1. Group averaged MEP-amplitudes (linearly detrended) for the Go condition for both condition sites. The best significant fitting cosine model for the r-preSMA condition is superimposed in red (20Hz) (A). Single subject MEP-amplitudes data (linearly detrended) for the Go trials when CS was delivered over the r-preSMA (B). Asterisks indicate *p*<0.05, graph bars represent mean standard errors.

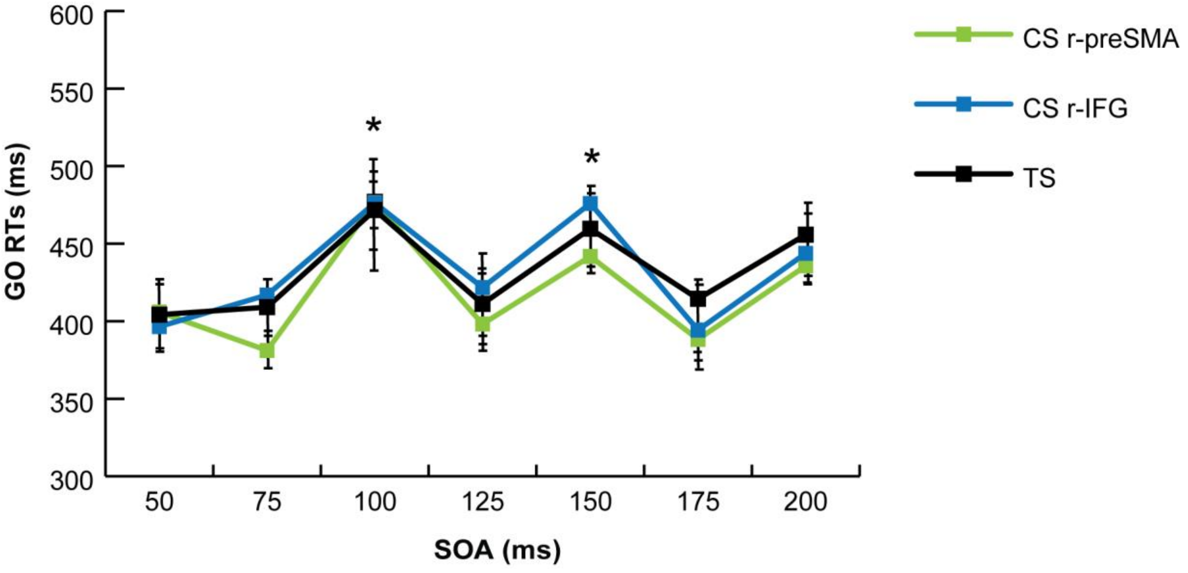

Figure S3 Related to Figure 1. Go RTs. RTs expressed in ms for TS alone, r-IFG or r-preSMA conditioning at different SOAs for Go trials. Asterisks indicates *p*<0.05. Graph bars represent mean standard errors.

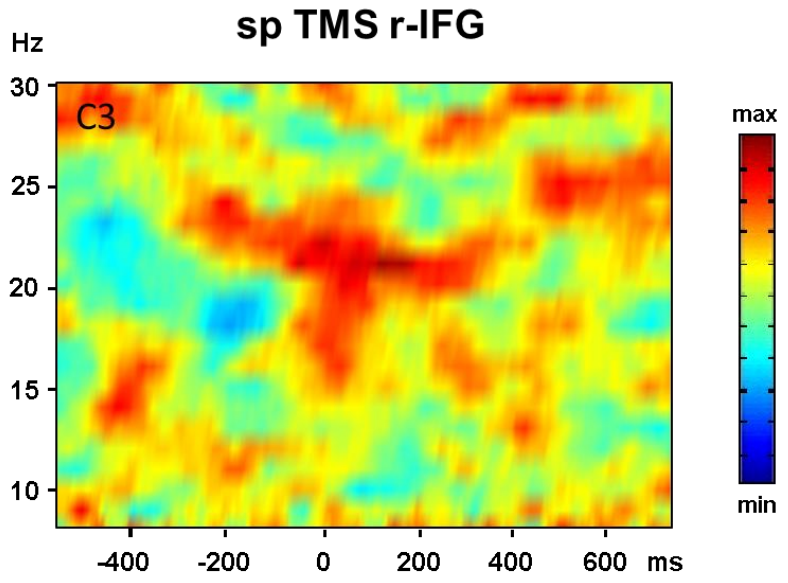

Figure S4 Related to Figure 4. Average Phase Locking Index (PLI), locked to the onset of the single TMS pulse applied over the r-IFG is displayed over time and frequency for the electrode closest to the left primary motor cortex (C3). The average PLI values calculated for the Go condition were subtracted from the values recorded during the Nogo condition. The difference is color coded according to the color bar on the right, and proved to be significant (see Supplemental Results below).

Supplemental Experimental Procedures and Methods

Participants

Seventeen healthy volunteers (9 women and 8 men; 24-31± years old) took part in this study. All subjects were right handed as assessed by the Edinburgh Handedness Inventory [S1]. All gave written informed consent for the study. The vision of all subjects was normal or corrected to normal. The experimental procedures were approved by the local Ethics Committee according to the Declaration of Helsinki.

Experimental setting

Subjects were seated in a comfortable armchair in a dimly illuminated, electrically shielded, and sound-proof room. Participants were instructed to keep their right index finger relaxed on a response key.

Go/Nogo task

Response inhibition was measured using a simple visual Go/Nogo paradigm trough Psyscope software. The present task is a modified version of a previous test used by Pandey *et al.* [S2]. The task entailed the presentation of four types of white isosceles triangles (cues) pointing upward, downward, rightward, or leftward against a black background. At the beginning of each trial a fixation cross was presented at the center of the computer screen for 500ms and after a blank of 200ms, the cue stimulus was shown for 100ms. The cue subtended a visual angle of approximately 1° (Figure 1B). In an initial training phase, participants were familiarized with the task until stable performance was reached. The training included a subset of trials, but all stimuli tested in the main experiment. The

experimental phase consisted of 128 stimuli, 32 of each type. Subjects were instructed to press a key with the right index finger whenever a white triangle pointed either up or down (Go stimulus) and refrain from pressing the key whenever the triangle pointed towards the right or left (Nogo stimulus). Subjects were instructed to respond as quickly as possible to the Go stimuli. The probability of occurrence of Go and Nogo stimuli was equal (50/50), and the order of stimulus presentation was pseudo-randomized. We choose this ratio in order to ensure a sufficient and equal number of MEPs recordings in all conditions (i.e. TS *vs.* CS+TS for every time interval and Go/Nogo condition). The inter-trial interval was 3000ms. Accuracy scores and reaction times (RTs) were recorded for each trial of the Go/Nogo task.

Paired pulse stimulation paradigm

Paired pulse stimulation was applied to investigate within a millisecond time scale the effective connectivity between r-IFG/l-M1 and r-preSMA/l-M1, by testing whether their activation could modulate M1 output as measured by MEP amplitude. The paired pulse stimulation technique was performed by means of two high-power Magstim 200 machines (Whitland, Dyfed, UK). The magnetic stimulus had a nearly monophasic pulse configuration, with a rise time of ~ 100μs, decaying back to zero over ~ 0.8ms. Electromyographic (EMG) traces were recorded from right FDI muscle by using 9-mm-in-diameter surface cup electrodes. The active electrode was placed over the muscle belly and the reference electrode over the metacarpophalangeal joint of the index finger. The ground electrode was placed over the right wrist. Responses were amplified with a Digitimer D360 amplifier through filters set at 20Hz and 2kHz, with a sampling rate of 5kHz and then recorded by a computer with the use of Signal software.

The Test Stimulus (TS) was applied over l-M1. In one half of the trials, l-M1 TMS was preceded by a conditioning stimulus (CS) delivered 6ms earlier over a contralateral prefrontal site (r-preSMA or r-IFG), or the control site (r-M1, control experiment). This inter-stimulus interval was selected according to recent studies performed with subjects at rest reporting relevant modulation of MEP amplitude following the application of a conditioning pulse on prefrontal sites and the application of a test stimulus on M1 spaced by 6ms [9, 10]. The intensity of TS was adjusted to evoke a MEP of ~ 1 mV peak to peak in the relaxed right FDI. The CS intensity was set at the 90% of the Resting Motor Threshold (RMT) [S3]. We defined RMT as the lowest intensity that evoked five small responses (~50µV) in the contralateral FDI muscle in a series of 10 stimuli in which the subject kept the FDI muscles relaxed in both hands [S4]. The test and the conditioning stimulator were connected to a small custom-made figure-of-eight-shaped coil (external diameter, 70mm) to reduce the effective area of stimulation. The coil over M1 was always placed tangentially to the scalp at the 45° angle from midline of the central sulcus, inducing a posterior-anterior current flow. The coil position for the r-IFG and for the r-preSMA was identified and monitored during all the experiments through the SofTaxic Navigator system (Electro Medical Systems) [S4, S5]. The r-IFG and r-preSMA were determined using individual structural MRI to localize the sites and adjusted with respect to individual sulcal landmarks in each participant. In order to target r-IFG, the coil was positioned over the caudal portion of the pars opercularis of the inferior frontal gyrus, handle pointing forward. The mean Talairach coordinates used in the present study to target r-IFG were x=52.9±3.1; y=9.6±14.5; z=19.4±3.3 (Brodmann area 44). In order to target r-preSMA, the coil was positioned over the dorso-medial frontal cortex close to the paracentral sulcus approximately in line with the vertical commissure anterior and was kept with the handle pointing laterally to induce a medially directed current in the

stimulated cortex. The mean Talairach coordinates used in the present study to target r-preSMA were x=1± 1.4; y=-13±6.4; z=60± 4.2 (Brodmann area 6)[S6].

Main Experiment

The conditioning sites (r-IFG and r-preSMA) were tested in two different sessions spaced 1 week apart in a pseudo-randomized order. Each session consisted of four blocks of 128 trials each. In these trials, TS alone or CS+TS TMS were applied during the fixation cross presentation (pre) or at 50, 75, 100, 125, 150, 175, 200ms after the visual cue onset with a number of 8 trials collected for each Go/Nogo condition and SOA. During each block, two different SOAs were randomly selected among all possible delays (pre, 50, 75, 100, 125, 150, 175, 200ms after cue presentation). The mean peak-to-peak amplitude of the conditioned MEP was expressed as a percentage of the mean peak-to-peak amplitude size of the unconditioned TS recorded at the same SOA.

Control Experiment

A control experiment was performed in a subsample of eight subjects to ensure that the conditioning effects found in the main experiment were not due to a spread of activation from r-IFG or r-preSMA to the right M1 (r-M1). The experiment was run in a single session consisting of one block of 128 trials and two selected SOAs (50 and 75ms) with a number of 8 trials collected for each Go/Nogo condition and SOA. The CS was delivered over r-M1, whereas the TS was applied over the l-M1 as for the main experiment. The mean peak-to-peak amplitude of the conditioned MEP was then expressed as a percentage of the mean peak-to-peak amplitude size of the unconditioned TS elicited at the same SOA.

The procedure was well tolerated by all subjects. The mean 1mV threshold was 48.5±8.1% for the main experiment and 47±7.4% for the control experiment. The stimulation intensity did not differ between the two experiments (t_{13}=0.7).

TMS-EEG Experiment

Seven additional healthy volunteers (4 women and 3 men; 25±3 years old) were enrolled in a combined TMS-EEG study. This experiment was planned to further investigate whether the difference between the Go *vs.* Nogo condition could be associated to changes in beta activity driven by r-IFG or r-preSMA activation. To this aim subjects were asked to perform the Go/Nogo task as for the main experiment while the EEG signal was continuously acquired. The experiment was run in a single session consisting of one block of 128 trials (64 Go trials and 64 Nogo trials) for each conditioning site (r-IFG, r-preSMA). For each trial a single TMS pulse was applied over r-IFG or r-preSMA at 150ms from cue onset. This SOA was selected based on the main experimental results, indicating a significant difference in conditioned MEP amplitude between Go and Nogo condition at a low variability across subjects. r-IFG and r-preSMA stimulation were performed in randomized order and separated by a TMS-free interval of ~15min to allow coil replacement. TMS intensity was adjusted to 90% of RMT to match the CS intensity used in the main study.

TMS-compatible EEG equipment (BrainAmp 32MRplus, BrainProducts GmbH, Munich, Germany) was used for recording EEG activity from the scalp [7]. The EEG was continuously acquired from 32 scalp sites positioned according to the 10-20 International System, using electrodes mounted on an elastic cap. Additional electrodes were used as ground and reference. The ground electrode was positioned in AFz, while an active reference was positioned on the tip of the nose. The signal was bandpass filtered at 0.1–1000Hz and

digitized at a sampling rate of 5kHz. In order to minimize overheating of the electrodes proximal to the stimulating coil, TMS-compatible Ag/AgCl sintered ring electrodes were used. Skin/electrode impedance was maintained below 5kΩ. Horizontal EOG (HEOG) was recorded from electrodes positioned on the outer canthi of both eyes and vertical EOG (VEOG) from electrodes located beneath the right eye recorded vertical eye movements and blinks. In order to reduce auditory contamination of EEG induced by coil clicks, subjects wore earplugs throughout the experiment.

Statistical Analyses TMS Experiments

For the main experiment conditioned MEP amplitude data were analyzed by a three-way repeated measures ANOVA with Site (r-IFG *vs.* r-pre-SMA), Condition (Go *vs.* Nogo) and SOA (pre, 50, 75, 100, 125, 150, 175, 200ms) as within-subject factors.

MEPs amplitude evoked by the TS without any CS were analyzed by a two-way repeated measures ANOVA with Condition (Go *vs.* Nogo) and SOA (pre, 50, 75, 100, 125, 150, 175, 200ms) as factors. Bonferroni corrected t-tests were performed when appropriate for all experiments.

As visual inspection and statistical analysis revealed an increased l-M1 excitability peaking every 50ms in the Nogo condition (see results section), we applied a curve-fitting procedure (robust nonlinear least-squares) in a Matlab custom made code to evaluate whether a cyclic pattern could be detected [S7]. Group-averaged conditioned MEPs amplitude acquired in the Nogo trials was linearly detrended to remove linear effects across SOA and retain any cyclic patterns around the mean. We then fitted to the r-IFG or r-preSMA-conditioned data all cosine curves from 1 to 40Hz. R-squared values of the group mean data were statistically evaluated using bootstrapping. To this end, labels of the 7 SOAs (from 50 to 200ms) were

randomly permuted over 2500 iterations, and a model cosine was fitted to the resulting MEP pattern each time, generating a null distribution of 2500 R-squared values for each cosine model. The R-squared value obtained from the actual data was related to this created null-distribution to evaluate whether it fell in the top-99th percentile, indicating that the model cosine significantly explained variance in the group data. Aside from the above-described group-mean analysis, we performed a secondary analysis, in which we fitted model cosines to the behavioral data of all individual participants on the single subject-level.

Despite no difference emerged during the Go trials between any of the intervals and the pre-cue condition (rest), indicating that any change in M1 excitability was not related to the task, but likely to a general connectivity properties between the prefrontal areas and M1, we decided to apply the same fitting procedure to the data acquired in the Go trials.

For the control experiment conditioned MEPs amplitude were analyzed by repeated measures ANOVA with factors Site (r-IFG, r-preSMA, r-M1), Condition (Go *vs.* Nogo) and SOA (50 *vs.* 75ms).

Behavioral data were analyzed by two-way repeated measures ANOVA with Site (CS r-IFG, conditioned stimulation over r-IFG, CS r-preSMA, conditioned stimulation over r-preSMA, TS, test stimulation alone) and SOA (50, 75, 100, 125, 150, 175 and 200ms) as within subjects factors performed on Go RTs.

Since fixed SOAs were chosen for the application of the TMS pulses Go RTs obtained following conditioned stimulation at 50, 100 and 150ms were correlated with the normalized MEP change at the same SOAs, separately for r-IFG and r-preSMA data, to check that the significant effects obtained in MEP data are independent of the specific phase of movement preparation in which each subject was.

EEG analysis and statistics

Preprocessing. EEG signals were first re-referenced to the average of all electrodes and high-pass filtered at 0.1Hz (Butterworth zero phase filter). For each subject, condition (Go *vs.* Nogo), and conditioning area (r-IFG *vs.* r-preSMA), pre-processing epochs were of 2s duration (-1s to + 1s from TMS pulse onset). Single trials were visually inspected to exclude epochs with excessively noisy EEG, eye movement artifacts, or muscle artifacts. Because of a low quality of the EEG recording, due to exceeding artifact contaminations, one subject had to be excluded from the study before post-processing. The artifact induced by pulse delivery, typically lasting 5–6ms with our equipment [S8], was removed using cubic interpolation for a conservative 10ms interval following the TMS pulse. Independent component analysis (ICA) was then run to identify and remove components reflecting residual muscle activity, eye movements, blink-related activity, and residual TMS-related artifacts (for details about ICs exclusion criteria see [9]). No more than four components were removed in each subject.

Time-frequency analysis. For each subject, condition and site, data were averaged from 1000ms before to 1000ms after pulse onset and then submitted to a complex Morlet wavelet (2–40Hz, 20 frequency steps, c=7). The frequency layers which significantly fitted (see fitting results) the MEPs modulation were then extracted from the wavelet dataset (central frequencies: 18.1 to 23.03Hz) for the electrodes closest to l-M1 (C3) and r-IPF (FC2) and r-SMA (FCz). The evoked activity over C3 was analyzed by means of repeated measure ANOVA with factors Condition (Go *vs.* Nogo) and Site of TMS (r-IFG *vs.* r-preSMA).

Source localization. Frequency analysis was performed for all clean trials collected during the r-IFG stimulation across all participants. Single trial data from a 250ms long window centered at latency 125ms was weighted with a Hanning window and subjected to FFT. The

cross spectral density matrix for all sensor combinations was computed for beta frequency at 20Hz both, for the Go and Nogo condition. Finally, the standard FieldTrip EEG volume conductor model was used together with the DICS beamformer on an 8mm grid to compute the spatial distribution of beta power for both conditions [S9].

Phase Locking Values. To investigate functional connectivity between r-IFG and l-M1 the phase locking value over time across all clean trials [S10] was computed. DICS beamformer coefficients were recomputed for a longer time window for the two target areas (for coordinates see Paired Pulse Stimulation paradigm above). Single trial data were bandpass filtered in the beta band and multiplied with beamformer coefficients to obtain time series of beta band activity for both regions of interest. Instantaneous phase was then computed via the Hilbert transform and the phase locking value was calculated for Nogo and Go condition by using the standard formula [S10]. The 99% confidence level for phase locking was computed by random permutation of single trials. The randomization was performed 1000 times. Finally, phase locking time series was plotted in units of standard deviation of the baseline [S11].

Phase Locking Index (PLI). To test if the cyclic pattern found for the MEP modulation in the Nogo condition could be explained by a local increase in beta phase consistency across trials, we computed PLI on artifact-free data for all frequencies from 2 to 30 Hz for the electrode closest to the left motor cortex (C3) (for single pulse TMS over the right IFG). To this end, data were submitted to a time frequency analysis (Hanning windowed) and PLI was then calculated for the Nogo and the Go condition separately by using the standard formula [S12]. The PLI values extracted for each condition were compared by means of a paired t-test.

Supplemental Results

MEP-measure of effective connectivity: single-subject data in Nogo trials

To verify whether the beta band models, which significantly fitted the group-averaged conditioned MEPs collected during the Nogo trials, also apply to individual data, we repeated the fitting procedure for each participant. When the CS was applied over the r-IFG, MEP amplitude modulations were significantly explained by a cosine model cycling at beta frequency for 9 (out of 10) participants (Figure S1A). More specifically, the same beta cosine models used for the group data (range 17-23Hz) also fitted the individual data of S1, S3-S4, S6-S8 and S10, while beta model ranges had to be slightly adapted for S2 (15-21Hz) and S5 (16-24Hz) to obtain significant fitting (bootstrapped 99%-cut-offs). No cosine model significantly explained MEPs data collected from S9.

When the CS was applied over the r-preSMA, single subject analysis revealed that the original cosine models in the beta-frequency range (from 17-23Hz) significantly fitted the MEP data of 8 (out of 10) participants (bootstrapped 99%-cut-off; Figure S1B). No significant fitting was obtained for S7 and S9.

MEP-measure of effective connectivity: group-averaged and single-subject data in Go trials

An additional fitting procedure was performed on group averaged conditioned MEPs data collected during the Go trial. Permutation tests revealed that cosine models in the beta-frequency range (from 19-21Hz) significantly fitted the MEP-data when the CS was delivered over r-preSMA (bootstrapped 99%-cut-off, 20Hz cosine being the model explaining the highest percentage of variance that is 66%; Figure S2A). No significant result emerged for

the r-IFG condition. However, when single subject data were subjected to the same fitting procedure, only data from 3 participants were significantly explained by the beta band cosine model (Figure S2B).

Performance measures (accuracy and RT) over SOAs in Go trials

In all conditions behavioural performance was highly accurate with low variability (accuracy Go trials: 99.3±1.1%; false alarms Nogo trials: 2.5±2.1%), also across conditions. For this reason both accuracy scores and false alarms RTs were not statistically analysed.

Analysis of reaction times (RT, two-way ANOVA on Go-trials only) showed a significant main effect of SOA ($F_{6,54}$=6.90; p<0.001). No other main effect or interaction reached significance. *Post-hoc* comparisons indicated that Go RTs were slowed down at 100 (all p <0.001) and 150 (all p<0.005) ms after the stimulus presentation with respect to the other SOAs, regardless of conditioning (Figure S3).

Performance measures: RT *vs.* MEP changes in Go trials

To probe whether the significant effects obtained in MEP data are independent of the specific phase of movement preparation in which each subject was, Go RTs obtained following paired-pulse stimulation at 50, 100 and 150ms (every second SOA) were correlated with the normalized MEP change at the same SOAs, separately for r-IFG and r-preSMA data. No significant correlation emerged (r-IFG SOA 50: r= -0.145; r-preSMA SOA 50: r=0.128; r-IFG SOA 100: r=0.274; r-preSMA SOA 100: r=-0.171; r-IFG SOA 150: r=-0.077; r-preSMA SOA 150 r=0.417; all p> 0.05).

Phase-locking index: Nogo vs Go condition

To test whether the oscillatory pattern found for the MEP amplitude cycling at beta frequency could reflect an increased inter-trial beta phase consistency across the Nogo- as

compared to the Go-trials, the PLI was calculated for both conditions and compared by means of a paired t-test. Figure S4 shows the difference between the two conditions (Nogo minus Go) when the TMS was applied over the r-IFG. Our results show that PLI was selectively increased around 20Hz in the Nogo trials. This increase was significant ($t(6)= 3.28$; $p=0.016$).

Supplemental References

S1. Oldfield, R.C. (1971). The assessment and analysis of handedness: the Edinburgh inventory. Neuropsychologia *9*, 97-113.

S2. Pandey, A.K., Kamarajan, C., Tang, Y., Chorlian, D.B., Roopesh, B.N., Manz, N., Stimus, A., Rangaswamy, M., and Porjesz, B. (2012). Neurocognitive deficits in male alcoholics: an ERP/sLORETA analysis of the N2 component in an equal probability Go/NoGo task. Biol Psychol. *89*, 170-182.

S3. Rossini, P.M., Barker, A.T., Berardelli, A., Caramia, M.D., Caruso, G., Cracco, R.Q., Dimitrijevic, M.R., Hallett, M., Katayama, Y., Lucking, C.H., et al. (1994). Non-invasive electrical and magnetic stimulation of the brain, spinal cord and roots: basic principles and procedures for routine clinical application. Report of an IFCN committee. Electroencephalogr Clin Neurophysiol. *91*, 79-92.

S4. Koch, G., Ponzo, V., Di Lorenzo, F., Caltagirone, C., and Veniero, D. (2013). Hebbian and anti-Hebbian spike-timing-dependent plasticity of human cortico-cortical connections. J Neurosci. *33*, 9725-9733.

S5. Koch, G., Cercignani, M., Pecchioli, C., Versace, V., Oliveri, M., Caltagirone, C., Rothwell, J., and Bozzali, M. (2010). In vivo definition of parieto-motor connections involved in planning of grasping movements. NeuroImage *51*, 300-312.

S6. Nachev, P., Kennard, C., and Husain, M. (2008). Functional role of the supplementary and pre-supplementary motor areas. Nat Rev Neurosci. *9*, 856-869.

S7. de Graaf, T.A., Gross, J., Paterson, G., Rusch, T., Sack, A.T., and Thut, G. (2013). Alpha-band rhythms in visual task performance: phase-locking by rhythmic sensory stimulation. PLoS One *8*, e60035.

S8. Veniero, D., Bortoletto, M., and Miniussi, C. (2009). TMS-EEG co-registration: on TMS-induced artifact. Clin Neurophysiol. *120*, 1392-1399.

S9. Gross, J., Kujala, J., Hamalainen, M., Timmermann, L., Schnitzler, A., and Salmelin, R. (2001). Dynamic imaging of coherent sources: Studying neural interactions in the human brain. Proc Natl Acad Sci U S A. 98, 694-9.

S10. Lachaux, J.P., Rodriguez, E., Martinerie, J., and Varela, F.J. (1999). Measuring phase synchrony in brain signals. Hum Brain Mapp. 8, 194-208.

S11. Rodriguez, E., George, N., Lachaux, J.P., Martinerie, J., Renault, B., and Varela, F.J. (1999). Perception's shadow: long-distance synchronization of human brain activity. Nature *397,* 430-3.

S12. Tallon-Baudry, C., Bertrand, O., Delpuech, C., Pernier, J. (1996). Stimulus Specificity of Phase-Locked and Non-Phase-Locked 40 Hz Visual Responses in Human. J Neurosci. *16*, 4240-4249.
