# Supplementary material for: Prefrontal Control over Motor Cortex Cycles at Beta Frequency during Movement Inhibition
Source: Curr Biol. 2014 Dec 15;24(24):2940–5. doi: 10.1016/j.cub.2014.10.043 (PMC4274313; doi:10.1016/j.cub.2014.10.043)
Supplement: Document S2. Article plus Supplemental Information [file mmc2.pdf]

Current Biology 24, 2940–2945, December 15, 2014 ©2014 The Authors http://dx.doi.org/10.1016/j.cub.2014.10.043

Report

Prefrontal Control over Motor Cortex Cycles at Beta Frequency during Movement Inhibition

Silvia Picazio,[1] Domenica Veniero,[1,2] Viviana Ponzo,[1] Carlo Caltagirone,[1,3] Joachim Gross,[2] Gregor Thut,[2] and Giacomo Koch[1,4,*]

[1]Non-Invasive Brain Stimulation Unit, Clinical and Behavioral Neurology Department, IRCCS Santa Lucia Foundation, Rome 00179, Italy

[2]Centre for Cognitive Neuroimaging, Institute of Neuroscience and Psychology, University of Glasgow, Glasgow G12 8QB, UK

[3]Department of System Medicine, Tor Vergata University, Rome 00133, Italy

[4]Stroke Unit, Department of Neuroscience, Policlinic Tor Vergata, Rome 00133, Italy

Summary

A fully adapted behavior requires maximum efficiency to inhibit processes in the motor domain [1]. Although a number of cortical and subcortical brain regions have been implicated, converging evidence suggests that activation of right inferior frontal gyrus (r-IFG) and right presupplementary motor area (r-preSMA) is crucial for successful response inhibition [2, 3]. However, it is still unknown how these prefrontal areas convey the necessary signal to the primary motor cortex (M1), the cortical site where the final motor plan eventually has to be inhibited or executed. On the basis of the widely accepted view that brain oscillations are fundamental for communication between neuronal network elements [4–6], one would predict that the transmission of these inhibitory signals within the prefrontal-central networks (i.e., r-IFG/M1 and/or r-preSMA/M1) is realized in rapid, periodic bursts coinciding with oscillatory brain activity at a distinct frequency. However, the dynamics of corticocortical effective connectivity has never been directly tested on such timescales. By using double-coil transcranial magnetic stimulation (TMS) and electroencephalography (EEG) [7, 8], we assessed instantaneous prefrontal-to-motor cortex connectivity in a Go/NoGo paradigm as a function of delay from (Go/NoGo) cue onset. In NoGo trials only, the effects of a conditioning prefrontal TMS pulse on motor cortex excitability cycled at beta frequency, coinciding with a frontocentral beta signature in EEG. This establishes, for the first time, a tight link between effective cortical connectivity and related cortical oscillatory activity, leading to the conclusion that endogenous (top-down) inhibitory motor signals are transmitted in beta bursts in large-scale cortical networks for inhibitory motor control.

Results

Double-Coil TMS Experiment

The real-time activity of right inferior frontal gyrus/left primary motor cortex (r-IFG/l-M1) and right presupplementary motor area/left primary motor cortex (r-preSMA/l-M1) connections was tested in healthy volunteers by double-coil transcranial magnetic stimulation (TMS) during the early response period of a simple visually cued Go/NoGo task (Figures 1A and 1B). To this end, we administered a conditioning stimulus (CS) over one of these right prefrontal areas followed by a test stimulus (TS) over left M1, always using a fixed CS-TS interstimulus interval of 6 ms (previously shown to probe instantaneous prefrontal-to-motor connectivity) [9, 10] (Figure 1A), but varying the delays of paired CS-TS TMS administration from the onset of the imperative (Go/NoGo) movement cue (stimulus onset asynchrony [SOA]: 50–200 ms, in steps of 25 ms) to probe for fluctuations of effective connectivity in these prefrontal/M1 networks over time (Figure 1B) during the execution of a Go/NoGo task [11]. We used motor-evoked potentials (MEPs) recorded from the first dorsal interosseus muscle of the right hand as dependent measure to verify the causal influence of conditioning pulses applied over either r-IFG or r-preSMA (for more information, see Supplemental Experimental Procedures available online). The experimental procedures were approved by the local Ethics Committee according to the Declaration of Helsinki.

Go/NoGo performance revealed a high accuracy rate in Go trials (99.3% ± 1.1%) and a low false alarm rate in NoGo trials (2.5% ± 2.1%), indicating good compliance of the participants. Reaction times (Go trials) were on average 428 ms (range across SOAs: 381–476 ms), indicating that all tested SOAs (50–200 ms) fell within an early premovement epoch corresponding to an early phase of motor planning.

In terms of cortical changes over time after Go/NoGo cueing, we first verified the time course of cortical excitability of l-M1 alone during the premovement period by analyzing MEP amplitude evoked by the TS without any CS. A main effect of SOA ($F_{7,63} = 2.67$; $p = 0.018$), but no significant modulation depending on the Go/NoGo condition or any interaction, was found for TS-induced MEP amplitude (Figure 1C). MEPs were reduced at 50 ms in comparison with all other SOAs, with the exception of the 200 ms SOA (all $p < 0.05$), indicating an unspecific decrease in l-M1 excitability immediately after the onset of the visual cue.

Testing r-IFG/l-M1 and r-preSMA/l-M1 effective connectivity by double-coil TMS (r-prefrontal CS/l-M1 TS) revealed a task-related temporal profile (Figures 1D and 1E), characterized by clear reverberant peaks of prefrontally enhanced l-M1 excitability at specific time points after the NoGo signal and a weaker (but counterphase) variation after the Go signal. Main effects of condition (Go versus NoGo) ($F_{1,9} = 9.66$; $p = 0.012$) and SOA ($F_{7,63} = 2.30$; $p = 0.037$) with a significant condition × SOA interaction ($F_{7,63} = 7.71$; $p < 0.001$) emerged on conditioned MEP amplitudes (Figure 1D). No effect of site (r-IFG versus r-preSMA) was found. MEPs were markedly and selectively increased for NoGo trials at 50 ms ($p = 0.003$), 100 ms ($p = 0.002$), and 150 ms ($p = 0.001$) after the stimulus onset in comparison to Go trials (Figure 1D). Importantly, when comparing these intervals to the conditioned MEP amplitude collected during the precue period, we found a significant difference at all these peaks in the NoGo condition (precue versus 50 ms: $p = 0.028$; precue versus 100 ms: $p = 0.027$; precue versus 150 ms: $p = 0.029$). This was not the case for the Go condition.

*Correspondence: g.koch@hsantalucia.it
This is an open access article under the CC BY license (http://creativecommons.org/licenses/by/3.0/).

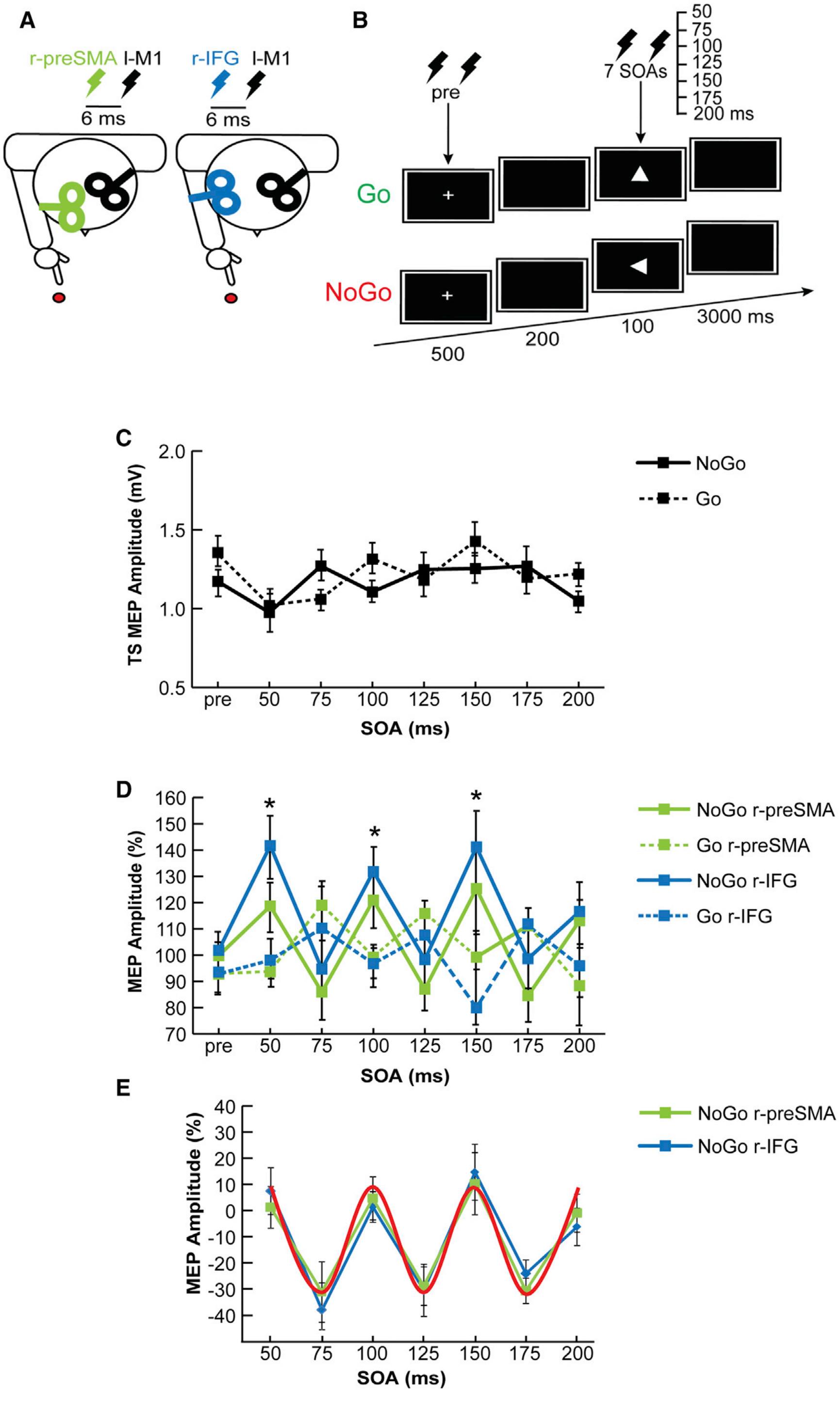

Figure 1. Experimental Setting and Results

(A) Schematic drawing of coil positioning on the head. A TMS pulse was applied over l-M1 (TS) either alone or proceeded by a CS (CS + TS) delivered 6 ms earlier over the r-IFG/r-preSMA.

(B) Single (TS) or paired pulse (CS + TS) TMSs were applied during the fixation cross presentation (pre) or during different delays (SOAs) after the presentation of the Go/NoGo cue during a premovement period (average RT was 428 ms). The task entailed the presentation of four types of white isosceles triangles pointing upward, downward, rightward, or leftward against a black background. Subjects were instructed to press a key with the right index finger whenever a white triangle pointed either up or down (Go stimulus) and to refrain from pressing the key whenever the triangle pointed toward the right or left (NoGo stimulus).

(C) MEP amplitude (mV) for TS alone for each CS site (r-IFG/r-preSMA) at different SOAs for Go and NoGo trials.

(D) MEP amplitude, expressed as percentage of change in comparison to TS alone, after r-IFG or r-preSMA conditioning at different SOAs for both Go and NoGo trials.

(E) Group-averaged MEP amplitudes (linearly detrended) for the NoGo condition. The best-fitting 20 Hz model cosine is superimposed (red line). Asterisks indicate $p < 0.01$. Here and elsewhere, graph bars represent mean SEs.

We then performed a curve-fitting procedure to examine whether the recurrently enhanced l-M1 excitability in the NoGo condition, peaking every 50 ms, can be accounted for by a cyclic pattern. Permutation tests revealed that cosine models in the beta-frequency range (from 17–23 Hz) significantly fitted the MEP data when the CS was delivered over r-IFG (20 Hz cosine being the best-fitting model; Figure 1E). The same results were found when the CS was applied over r-preSMA (best fit: 20 Hz cosine; Figure 1E). This supports the existence of a beta-oscillation underling group-averaged MEP amplitude modulation during NoGo trials, as tested by prefrontal conditioning. Crucially, cosine fitting in the beta range was also significant at the individual level (as confirmed by single-subject analysis of the MEP measure of effective connectivity) for NoGo data, but not for Go data (see Supplemental Results and Figures S1 and S2).

We conducted a control experiment exploring r-M1/l-M1 connectivity (Figure 2A) to ensure that the results obtained when the CS was applied over the r-IFG or r-preSMA were due to the conditioning effect of the pulse over the targeted prefrontal areas and not due to a spread of activation to nearby r-M1. We chose two SOAs (50 ms versus 75 ms) showing significant variations in inferred connectivity in the main experiment (i.e., r-IFG/l-M1 and r-preSMA/l-M1). r-M1/l-M1 connectivity did not show a similar modulation of corticospinal excitability as r-IFG/l-M1 and r-preSMA/l-M1 probes did (main effect of site: $F_{2,14} = 6.79$; $p = 0.008$) (Figure 2B).

TMS-EEG Experiment

We conducted a TMS-electroencephalography (EEG) experiment to directly probe instantaneous oscillatory network activity through the administration of a single TMS pulse over r-IFG or r-preSMA at 150 ms into the execution of the Go/NoGo paradigm while simultaneously recording EEG [8, 12, 13]. This 150 ms SOA was selected on the basis of the findings

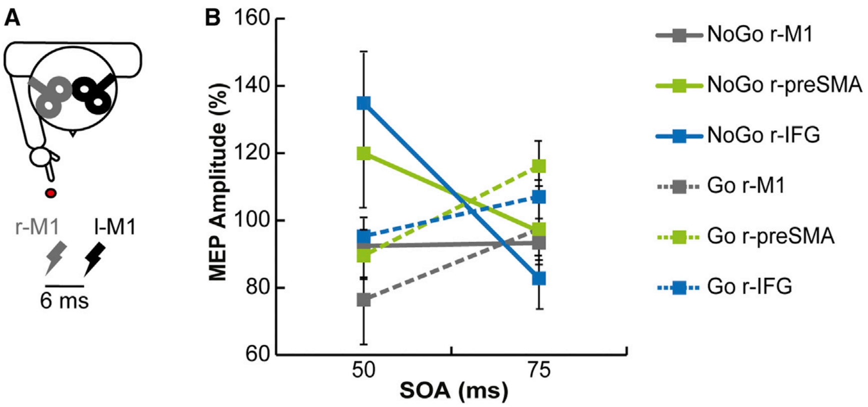

Figure 2. Experimental Setting and Results of the Control Experiment

(A) Schematic drawing of coil positioning on the head. TMS was applied either alone over l-M1 or proceeded by a CS delivered 6 ms earlier over the r-M1, r-IFG, or r-preSMA at two different delays (50 ms and 75 ms) after the onset of the Go/NoGo cue.

(B) MEP amplitude, expressed as percentage of change in comparison to TS alone, after r-M1, r-IFG, or r-preSMA conditioning at different SOAs for both Go and NoGo trials.

obtained in the double-coil TMS experiment. Figure 3A shows a time-frequency plot after subtraction of the evoked activity recorded in the NoGo condition minus Go condition for the two electrodes close to the (conditioning) TMS sites (FC2 for r-IFC and FCz for r-preSMA) and for the electrode closest to l-M1 (C3). This demonstrates dominant sustained beta increases (20–23 Hz peak frequency, 50–250 ms after cue onset), starting at early time points over prefrontal sites and spreading to left central sites after both r-IFG and r-preSMA stimulation in NoGo as compared to Go trials (Figure 3A). In line with the MEP results, the EEG topography (after 150 ms) revealed a right frontal-left central signature (Figure 3A, map inset). A main effect of condition (Go versus NoGo) ($F_{1,5} = 15.8$; $p = 0.01$) was found for C3 beta power, with higher beta power during the NoGo condition than the Go condition (Figure 3B). No effect of site was found on C3 beta activity, further suggesting that r-IFG and r-preSMA activation can result in an M1 excitability modulation by acting on the same oscillatory activity. This therefore links the cyclic pattern of prefrontal-M1 connectivity in the beta range as revealed by double-coil TMS to oscillatory brain activity as recorded by EEG (NoGo > Go), without being able to resolve where the NoGo-related surface EEG activity at beta frequency is generated (i.e., whether it is predominantly restricted to M1 or prefrontal areas, or whether it is reflecting network activity).

To address this point, we performed additional analyses in source space (see Supplemental Experimental Procedures). Given the restrictions of the EEG recording (i.e., limited number of electrodes and its low spatial resolution), we limited the source analysis to the IFG condition (with M1 and preSMA not being separable because of their proximity). The source analysis that was performed to identify the generator of beta activity evoked by TMS over r-IFG during the NoGo trials revealed left M1 as a prominent source (Figure 4A). To investigate functional connectivity between this area and r-IFG, we calculated phase-locking value in the beta band for Go and NoGo trials in the r-IFG condition (see Supplemental Experimental Procedures). As shown in Figure 4B, a significant phase coherence increase between the two areas was present for the NoGo, but not the Go, condition, and this occurred during the relevant time interval (around 50–150 ms). Hence, cyclic patterns in prefrontal-M1 connectivity in the beta range (NoGo > Go), as inferred from double-coil TMS, not only coincide with EEG beta increases over areas of this network but also can be linked to more direct EEG measures of functional network activity in this frequency band.

Finally, we calculated the phase-locking index (PLI) across trials for the electrode closest to M1 (C3) to test whether the activation of r-IFG realigns the instantaneous beta phase over the motor cortex. This analysis revealed that the NoGo condition was characterized by a significantly increased intertrial phase consistency in the beta range when compared to the Go condition (see Supplemental Results and Figure S4).

Discussion

The current findings provide first-time evidence linking reverberations in effective strength of cortical connectivity with underlying cortical oscillations in the motor system. The early causal inhibitory influences of prefrontal cortex on M1 activity are not transferred in a discrete manner but follow an oscillatory beta rhythm. Notably, this coincides with the presence of beta oscillations in the prefrontal-motor pathway, as revealed by TMS-EEG experiments. These results imply that the transmission of causal influences in cortical networks is related to channels of communication tuned to specific frequencies, here in the beta rhythm. The findings provide important information on the implication of oscillations in brain function in general, namely on the open question of to what extent cortical rhythms underlie network communication per se. Specifically, they also provide novel information on the causal implication of beta rhythms in cortical inhibitory motor control.

There is accumulating evidence for a causal implication of brain oscillations [14]. Several recent behavioral studies [15–17] unveiled rhythmic fluctuations in performance measures after temporal reset of visual or attentional processing due to the presentation of a discrete sensory event (see [18] for a review). This coincided with the periodicity of concurrently recorded rhythms of the visual brain, revealed by simultaneous EEGs [17]. Similar to the above studies [15–17], our data set examines cycles in behavioral measures as a function of delay from an external event (here, the imperative Go/NoGo signal). Here, we tested the instantaneous communication between two brain areas of a network at different time points, unlike the above studies, which tested visual performance (and hence excitability) at a single time point. By showing that this measure of instantaneous effective connectivity cycles at beta frequency and coincides with beta oscillation, we demonstrate in novel ways that cortical connectivity per se relates to rhythms of the brain. Importantly, no such cycles were observed with single-pulse TMS over motor cortex following the presentation of the NoGo cue, ruling out an explanation of fluctuating motor cortex excitability in our case. In addition, we did not find motor cortex excitability as

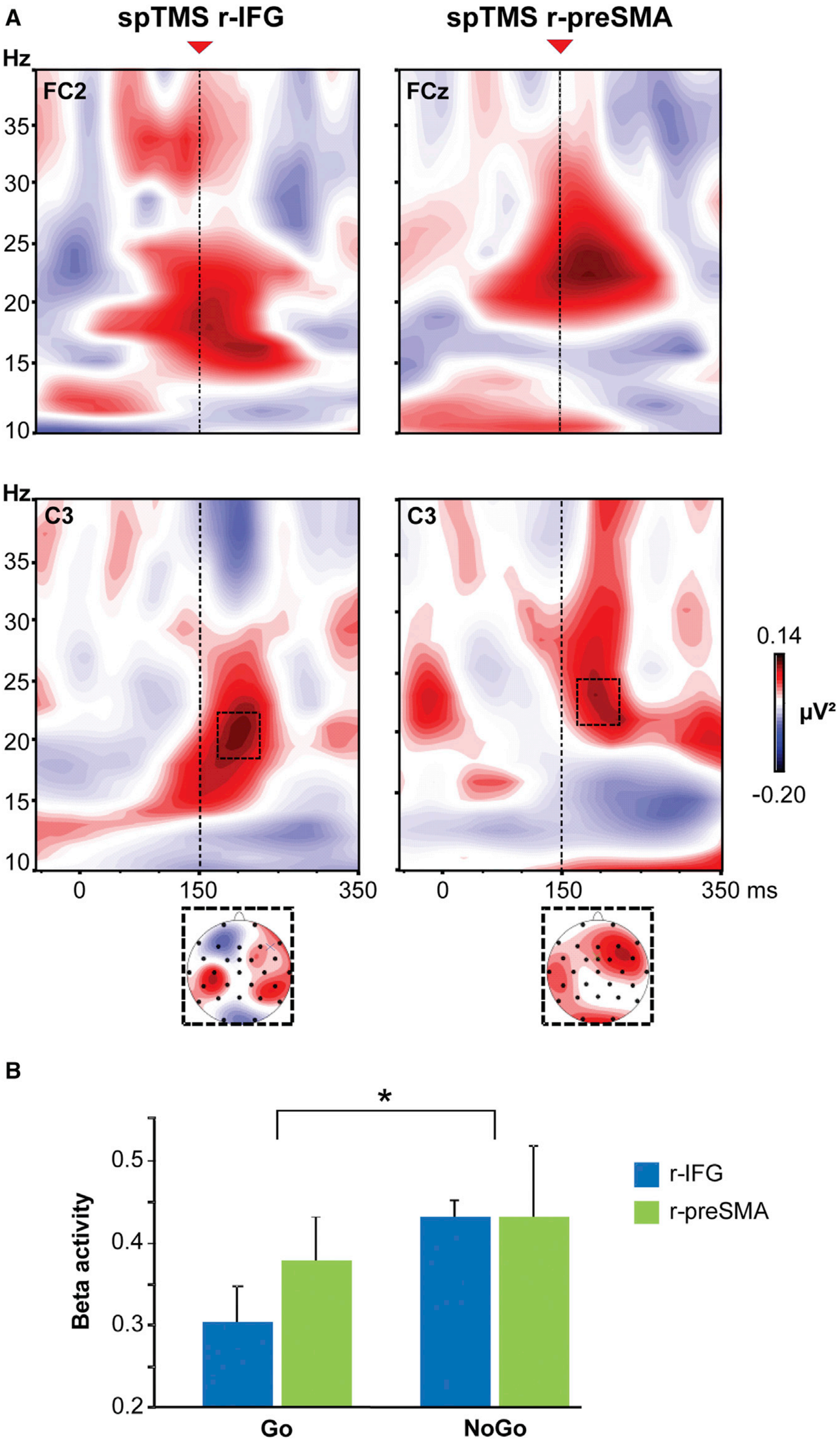

Figure 3. TMS-EEG Experiment Results

(A) Time-frequency plots for each TMS site (r-IFG on the left, r-preSMA on the right) are shown for C3 and for two electrodes close to the conditioning site. The activity recorded for the Go condition was subtracted from the activity recorded during the NoGo condition. Zero represents time of visual cue onset, whereas the red arrows indicate the timing of pulse delivery (150 ms after cue onset). The dotted squares indicate a significant difference between conditions and the corresponding topographical map.

(B) Beta power plotted as function of condition and TMS site. Asterisk indicates $p < 0.05$.

tested by single-pulse TMS to inversely relate to beta power, i.e., excitability was not globally reduced in the NoGo condition for which higher beta power was observed (Figure 1C). Instead, when the CS was applied over prefrontal areas, MEP amplitudes were enhanced at 50 ms, 100 ms, and 150 ms after the NoGo cue but showed a trend of being reduced at 75 ms, 125 ms, and 175 ms after the NoGo cue (Figure 1D). Therefore, despite the fact that the required final output was inhibiting the action, we found an alternating pattern of increasing and decreasing MEP amplitudes at specific time intervals in an early phase of motor planning (0–200 ms after Go/NoGo cueing). This points to beta phase playing a functional role in this interval rather than beta amplitude, and it implies underlying changes in primary motor cortex connectivity rather than in excitability, as further confirmed by the phase-locking results.

Our results therefore provide direct support for the suggestion that brain oscillations are related to communications within neural networks [4–6]. In particular, they are in accord with the views that synchronization of oscillatory activity reflects spectral fingerprints of large-scale neuronal interactions [5, 19] linking beta oscillations with a large-scale inhibitory motor control network [20, 21] and that communication in these networks occurs in bursts at their typical frequencies, as predicted by the communication-through-coherence theory [6, 22]. The current results are also in line with the findings that beta oscillations seem prominently involved in top-down (feedback) interactions within cortical networks, as compared to higher frequency oscillations in the gamma band [5, 23, 24], and with a recent TMS-EEG study showing that the inferior prefrontal cortex has specific resonant properties in the beta-frequency range [25]. Here, we show for the first time that spectral EEG fingerprints of top-down inhibitory control signals are also visible in direct measures of corticocortical interactions of the corresponding network (tested by double-coil TMS). The main interpretation of our results is therefore that functional coupling of the prefrontal areas with the motor cortex in NoGo conditions occurs at a beta rhythm. We excluded that the NoGo cue induced similar oscillation in local l-M1 excitability independently from prefrontal input because in the control experiment, r-M1 pulses did not elicit rhythmicity in l-M1 MEPs under otherwise identical conditions. However, we cannot infer based on

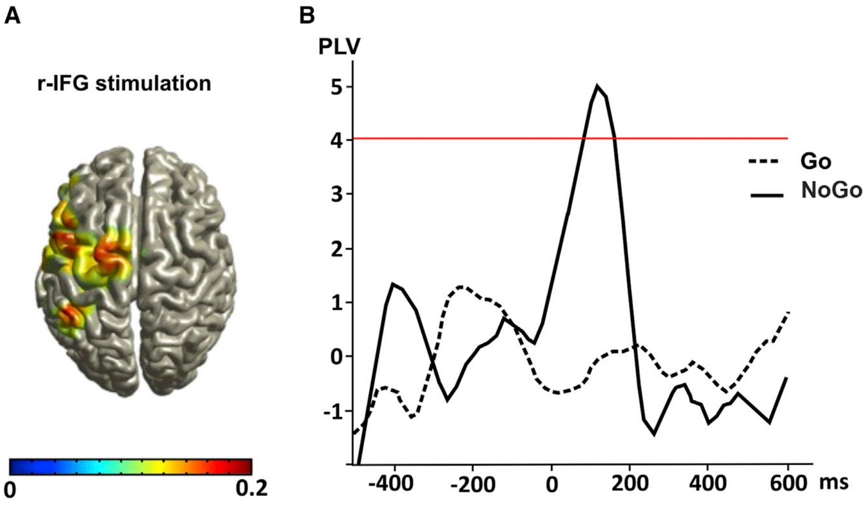

Figure 4. Source Analysis and PLV

(A) Estimate of most prominent beta sources calculated for a 100 ms time window after the TMS pulse on the relative changes between the NoGo and Go condition.

(B) Phase-locking time series, plotted in units of SD, of the baseline for the Go and NoGo condition. The horizontal red line indicates the 99% confidence level.

our data how this communication comes about. It is possible that beta oscillations, which are prominently represented in both areas, become phase aligned, opening the communication between these areas. Another possibility is that the NoGo cues directly induce beta oscillation in the prefrontal areas and that the periodicity in the connected motor cortex is a natural consequence of this prefrontal oscillation (as TMS pulses in connected M1 may be more potent when applied in coincidence with up states of excitability in right frontal cortex). Nonetheless, both scenarios go along with prefrontal areas showing cyclic changes in strength of coupling (i.e., effective connectivity) at beta frequency.

In terms of the anatomy of inhibitory motor control, the interactions of both the r-IFG and the r-preSMA with the left M1 revealed a similar temporal profile during NoGo trials. This result is in accordance with increasing evidence indicating that both areas are crucial for response inhibition, albeit with a possible lateralization to the right hemisphere, suggesting that these two regions work together to exert a causal control in the early phases of movement inhibition [2, 3]. Although no clear difference between r-IFG and r-preSMA emerged, it has to be noted that the beta-power increase in the NoGo condition, which was found during the TMS-EEG experiment, was predominantly due to the modulation caused by the r-IFG TMS (Figure 3B). Another limitation of the present study is the sampling rate used to acquire the MEP data (40 Hz). As a consequence, we cannot exclude that frequencies higher than beta are involved in the coupling between the prefrontal areas and the primary motor cortex or that the results found are due to aliasing of higher frequencies, although the EEG results covering a bigger frequency range do point to the same frequencies, i.e., beta frequency.

In terms of rhythms of the motor system, our TMS-EEG data link the findings of reverberant connectivity at beta frequency to central beta-band activity that has traditionally been viewed to reflect an idling state of motor cortices [26], supported by the findings that beta activity is attenuated during voluntary movements. However, accumulating evidence indicates that beta-band activity is causally involved in motor control [27, 28] and actively engaged in promoting the existing motor state and preventing new movement initiation [29]. In addition to inhibitory control of action by beta oscillation, recent evidence also points toward inhibitory control by means of alpha oscillations [21, 30]. These findings are not mutually exclusive because here, we focus on the causal influence of prefrontal areas on motor cortex excitability (i.e., corticocortical connectivity), whereas the former studies focused on upper alpha activity at sensorimotor cortices and corticospinal activity per se [30]. Taken together, our data reveal for the first time that beta activity reflects bursts of top-down, corticocortical network interactions serving a role in premovement inhibition of motor acts. This is more in line with the hypothesis that beta oscillations may predominantly reflect endogenously driven processes serving the maintenance of the status quo of a current sensory-motor or cognitive state [31] than the initial idling-state hypothesis.

Conclusions

Our work provides novel evidence for a link between effective cortical connectivity and related cortical oscillatory activity in mediating response inhibition that has not been observed before. These findings could have important implications in unraveling abnormalities of motor and cognitive inhibition in several pathological conditions, including Parkinson's disease, autism, and schizophrenia [32, 33], and could be important in building novel therapeutic strategies based on invasive or noninvasive cortical stimulation or neuroprosthesis.

Supplemental Information

Supplemental Information includes Supplemental Results, Supplemental Experimental Procedures, and four figures and can be found with this article online at http://dx.doi.org/10.1016/j.cub.2014.10.043.

Author Contributions

S.P. and G.K. conceived the study. S.P. and D.V. performed the experiments and data analysis. J.G. performed data analysis. V.P. performed the experiments. C.C., G.T., and G.K. supervised the experiments. S.P., D.V., J.G., C.C., G.T., and G.K. wrote the paper.

Acknowledgments

This work was supported by a grant from the Italian Ministry of Health to G.K. (239/GR-2009-1591859) and by a Wellcome Trust Joint Investigator Award to G.T. and J.G. (098434 and 098433).

Received: May 5, 2014
Revised: September 4, 2014
Accepted: October 14, 2014
Published: December 4, 2014

References

1. Aron, A.R., Durston, S., Eagle, D.M., Logan, G.D., Stinear, C.M., and Stuphorn, V. (2007). Converging evidence for a fronto-basal-ganglia

network for inhibitory control of action and cognition. J. Neurosci. *27*, 11860–11864.
2. Shibasaki, H. (2012). Cortical activities associated with voluntary movements and involuntary movements. Clin. Neurophysiol. *123*, 229–243.
3. Swann, N.C., Cai, W., Conner, C.R., Pieters, T.A., Claffey, M.P., George, J.S., Aron, A.R., and Tandon, N. (2012). Roles for the pre-supplementary motor area and the right inferior frontal gyrus in stopping action: electrophysiological responses and functional and structural connectivity. Neuroimage *59*, 2860–2870.
4. Schnitzler, A., and Gross, J. (2005). Normal and pathological oscillatory communication in the brain. Nat. Rev. Neurosci. *6*, 285–296.
5. Siegel, M., Donner, T.H., and Engel, A.K. (2012). Spectral fingerprints of large-scale neuronal interactions. Nat. Rev. Neurosci. *13*, 121–134.
6. Fries, P. (2005). A mechanism for cognitive dynamics: neuronal communication through neuronal coherence. Trends Cogn. Sci. *9*, 474–480.
7. Koch, G., Franca, M., Del Olmo, M.F., Cheeran, B., Milton, R., Alvarez Sauco, M., and Rothwell, J.C. (2006). Time course of functional connectivity between dorsal premotor and contralateral motor cortex during movement selection. J. Neurosci. *26*, 7452–7459.
8. Veniero, D., Ponzo, V., and Koch, G. (2013). Paired associative stimulation enforces the communication between interconnected areas. J. Neurosci. *33*, 13773–13783.
9. Arai, N., Müller-Dahlhaus, F., Murakami, T., Bliem, B., Lu, M.K., Ugawa, Y., and Ziemann, U. (2011). State-dependent and timing-dependent bidirectional associative plasticity in the human SMA-M1 network. J. Neurosci. *31*, 15376–15383.
10. Bäumer, T., Schippling, S., Kroeger, J., Zittel, S., Koch, G., Thomalla, G., Rothwell, J.C., Siebner, H.R., Orth, M., and Münchau, A. (2009). Inhibitory and facilitatory connectivity from ventral premotor to primary motor cortex in healthy humans at rest—a bifocal TMS study. Clin. Neurophysiol. *120*, 1724–1731.
11. Waldvogel, D., van Gelderen, P., Muellbacher, W., Ziemann, U., Immisch, I., and Hallett, M. (2000). The relative metabolic demand of inhibition and excitation. Nature *406*, 995–998.
12. Capotosto, P., Babiloni, C., Romani, G.L., and Corbetta, M. (2009). Frontoparietal cortex controls spatial attention through modulation of anticipatory alpha rhythms. J. Neurosci. *29*, 5863–5872.
13. Massimini, M., Ferrarelli, F., Huber, R., Esser, S.K., Singh, H., and Tononi, G. (2005). Breakdown of cortical effective connectivity during sleep. Science *309*, 2228–2232.
14. Thut, G., Miniussi, C., and Gross, J. (2012). The functional importance of rhythmic activity in the brain. Curr. Biol. *22*, R658–R663.
15. Landau, A.N., and Fries, P. (2012). Attention samples stimuli rhythmically. Curr. Biol. *22*, 1000–1004.
16. Fiebelkorn, I.C., Saalmann, Y.B., and Kastner, S. (2013). Rhythmic sampling within and between objects despite sustained attention at a cued location. Curr. Biol. *23*, 2553–2558.
17. Romei, V., Gross, J., and Thut, G. (2012). Sounds reset rhythms of visual cortex and corresponding human visual perception. Curr. Biol. *22*, 807–813.
18. VanRullen, R., and Koch, C. (2003). Is perception discrete or continuous? Trends Cogn. Sci. *7*, 207–213.
19. Hipp, J.F., Hawellek, D.J., Corbetta, M., Siegel, M., and Engel, A.K. (2012). Large-scale cortical correlation structure of spontaneous oscillatory activity. Nat. Neurosci. *15*, 884–890.
20. Zhang, Y., Chen, Y., Bressler, S.L., and Ding, M. (2008). Response preparation and inhibition: the role of the cortical sensorimotor beta rhythm. Neuroscience *156*, 238–246.
21. Sauseng, P., Gerloff, C., and Hummel, F.C. (2013). Two brakes are better than one: the neural bases of inhibitory control of motor memory traces. Neuroimage *65*, 52–58.
22. Womelsdorf, T., Schoffelen, J.M., Oostenveld, R., Singer, W., Desimone, R., Engel, A.K., and Fries, P. (2007). Modulation of neuronal interactions through neuronal synchronization. Science *316*, 1609–1612.
23. Siegel, M., Körding, K.P., and König, P. (2000). Integrating top-down and bottom-up sensory processing by somato-dendritic interactions. J. Comput. Neurosci. *8*, 161–173.
24. Buschman, T.J., and Miller, E.K. (2007). Top-down versus bottom-up control of attention in the prefrontal and posterior parietal cortices. Science *315*, 1860–1862.
25. Hanslmayr, S., Matuschek, J., and Fellner, M.C. (2014). Entrainment of prefrontal beta oscillations induces an endogenous echo and impairs memory formation. Curr. Biol. *24*, 904–909.
26. Pfurtscheller, G., Stancák, A., Jr., and Neuper, C. (1996). Event-related synchronization (ERS) in the alpha band—an electrophysiological correlate of cortical idling: a review. Int. J. Psychophysiol. *24*, 39–46.
27. Joundi, R.A., Jenkinson, N., Brittain, J.S., Aziz, T.Z., and Brown, P. (2012). Driving oscillatory activity in the human cortex enhances motor performance. Curr. Biol. *22*, 403–407.
28. Pogosyan, A., Gaynor, L.D., Eusebio, A., and Brown, P. (2009). Boosting cortical activity at Beta-band frequencies slows movement in humans. Curr. Biol. *19*, 1637–1641.
29. Gilbertson, T., Lalo, E., Doyle, L., Di Lazzaro, V., Cioni, B., and Brown, P. (2005). Existing motor state is favored at the expense of new movement during 13-35 Hz oscillatory synchrony in the human corticospinal system. J. Neurosci. *25*, 7771–7779.
30. Sauseng, P., Klimesch, W., Gerloff, C., and Hummel, F.C. (2009). Spontaneous locally restricted EEG alpha activity determines cortical excitability in the motor cortex. Neuropsychologia *47*, 284–288.
31. Engel, A.K., and Fries, P. (2010). Beta-band oscillations—signalling the status quo? Curr. Opin. Neurobiol. *20*, 156–165.
32. Juan, C.H., and Muggleton, N.G. (2012). Brain stimulation and inhibitory control. Brain Stimulat. *5*, 63–69.
33. Obeso, I., Wilkinson, L., Casabona, E., Bringas, M.L., Álvarez, M., Álvarez, L., Pavón, N., Rodríguez-Oroz, M.C., Macías, R., Obeso, J.A., and Jahanshahi, M. (2011). Deficits in inhibitory control and conflict resolution on cognitive and motor tasks in Parkinson’s disease. Exp. Brain Res. *212*, 371–384.

Current Biology, Volume *24*

Supplemental Information

Prefrontal Control over Motor Cortex Cycles at Beta Frequency during Movement Inhibition

Silvia Picazio, Domenica Veniero, Viviana Ponzo, Carlo Caltagirone, Joachim Gross, Gregor Thut, and Giacomo Koch

Supplemental Figure and Legends

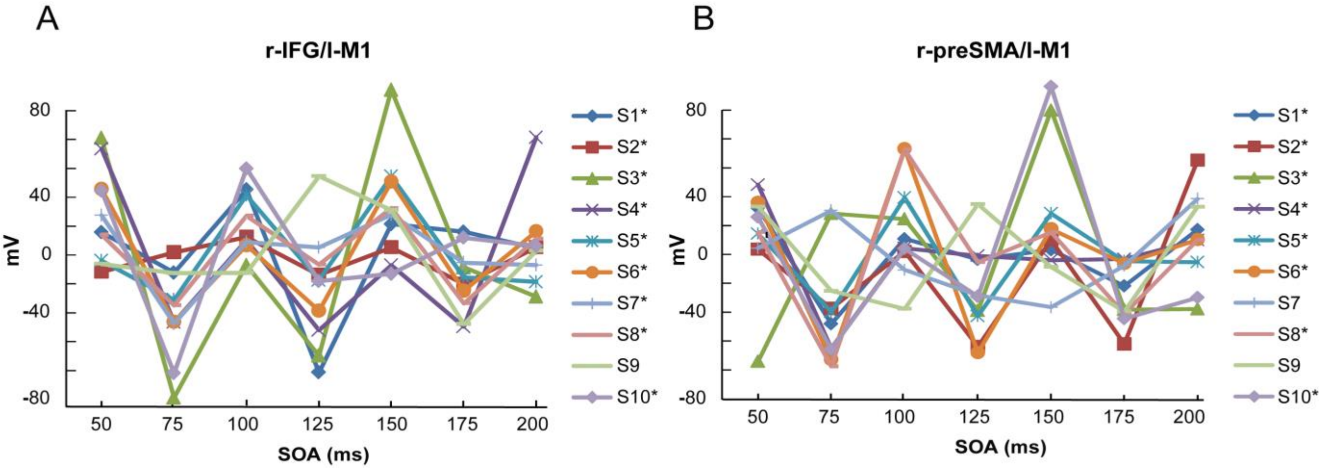

Figure S1 Related to Figure 1. Single-subject MEP-amplitudes data (linearly detrended) for the Nogo trials for the two conditioning sites, r-IFG (A) and r-preSMA (B). Asterisks indicates a significant beta band fitting (*p*<0.05).

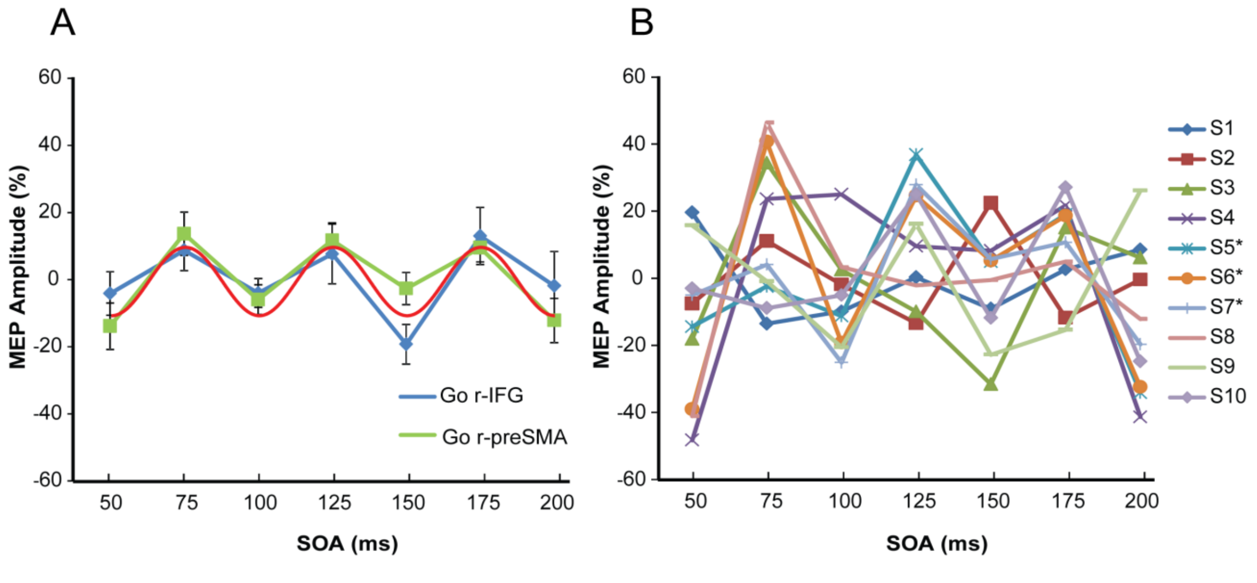

Figure S2 Related to Figure 1. Group averaged MEP-amplitudes (linearly detrended) for the Go condition for both condition sites. The best significant fitting cosine model for the r-preSMA condition is superimposed in red (20Hz) (A). Single subject MEP-amplitudes data (linearly detrended) for the Go trials when CS was delivered over the r-preSMA (B). Asterisks indicate *p*<0.05, graph bars represent mean standard errors.

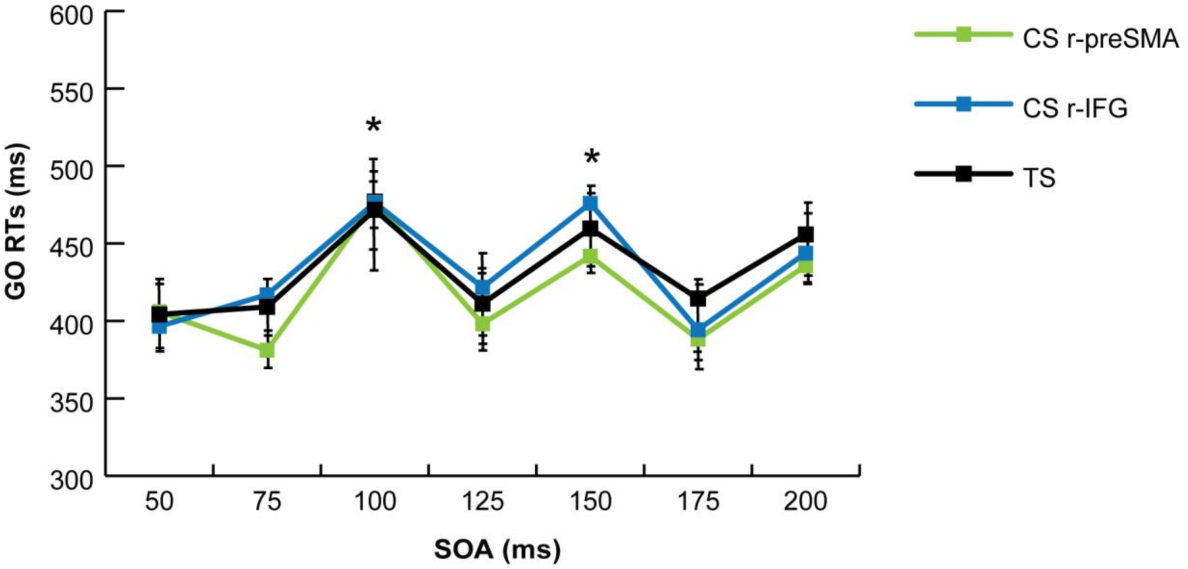

Figure S3 Related to Figure 1. Go RTs. RTs expressed in ms for TS alone, r-IFG or r-preSMA conditioning at different SOAs for Go trials. Asterisks indicates *p*<0.05. Graph bars represent mean standard errors.

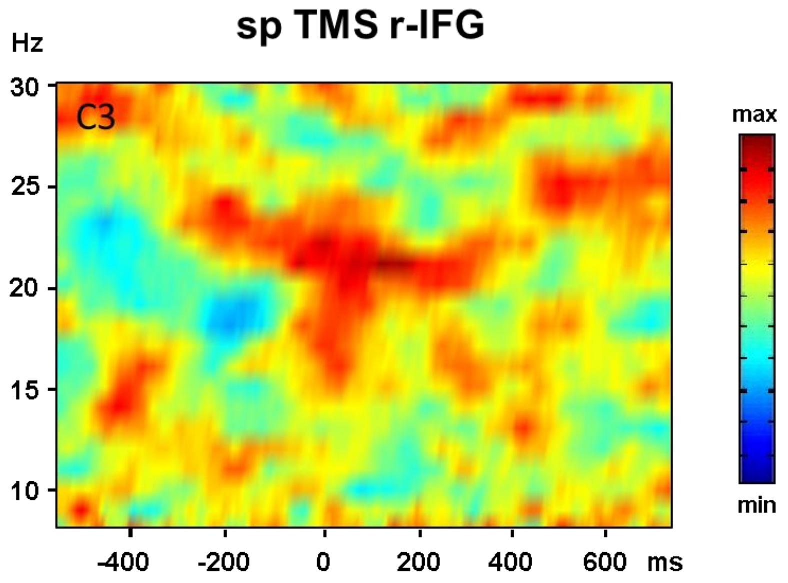

Figure S4 Related to Figure 4. Average Phase Locking Index (PLI), locked to the onset of the single TMS pulse applied over the r-IFG is displayed over time and frequency for the electrode closest to the left primary motor cortex (C3). The average PLI values calculated for the Go condition were subtracted from the values recorded during the Nogo condition. The difference is color coded according to the color bar on the right, and proved to be significant (see Supplemental Results below).

Supplemental Experimental Procedures and Methods

Participants

Seventeen healthy volunteers (9 women and 8 men; 24-31± years old) took part in this study. All subjects were right handed as assessed by the Edinburgh Handedness Inventory [S1]. All gave written informed consent for the study. The vision of all subjects was normal or corrected to normal. The experimental procedures were approved by the local Ethics Committee according to the Declaration of Helsinki.

Experimental setting

Subjects were seated in a comfortable armchair in a dimly illuminated, electrically shielded, and sound-proof room. Participants were instructed to keep their right index finger relaxed on a response key.

Go/Nogo task

Response inhibition was measured using a simple visual Go/Nogo paradigm trough Psyscope software. The present task is a modified version of a previous test used by Pandey *et al.* [S2]. The task entailed the presentation of four types of white isosceles triangles (cues) pointing upward, downward, rightward, or leftward against a black background. At the beginning of each trial a fixation cross was presented at the center of the computer screen for 500ms and after a blank of 200ms, the cue stimulus was shown for 100ms. The cue subtended a visual angle of approximately 1° (Figure 1B). In an initial training phase, participants were familiarized with the task until stable performance was reached. The training included a subset of trials, but all stimuli tested in the main experiment. The

experimental phase consisted of 128 stimuli, 32 of each type. Subjects were instructed to press a key with the right index finger whenever a white triangle pointed either up or down (Go stimulus) and refrain from pressing the key whenever the triangle pointed towards the right or left (Nogo stimulus). Subjects were instructed to respond as quickly as possible to the Go stimuli. The probability of occurrence of Go and Nogo stimuli was equal (50/50), and the order of stimulus presentation was pseudo-randomized. We choose this ratio in order to ensure a sufficient and equal number of MEPs recordings in all conditions (i.e. TS *vs.* CS+TS for every time interval and Go/Nogo condition). The inter-trial interval was 3000ms. Accuracy scores and reaction times (RTs) were recorded for each trial of the Go/Nogo task.

Paired pulse stimulation paradigm

Paired pulse stimulation was applied to investigate within a millisecond time scale the effective connectivity between r-IFG/l-M1 and r-preSMA/l-M1, by testing whether their activation could modulate M1 output as measured by MEP amplitude. The paired pulse stimulation technique was performed by means of two high-power Magstim 200 machines (Whitland, Dyfed, UK). The magnetic stimulus had a nearly monophasic pulse configuration, with a rise time of ~ 100μs, decaying back to zero over ~ 0.8ms. Electromyographic (EMG) traces were recorded from right FDI muscle by using 9-mm-in-diameter surface cup electrodes. The active electrode was placed over the muscle belly and the reference electrode over the metacarpophalangeal joint of the index finger. The ground electrode was placed over the right wrist. Responses were amplified with a Digitimer D360 amplifier through filters set at 20Hz and 2kHz, with a sampling rate of 5kHz and then recorded by a computer with the use of Signal software.

The Test Stimulus (TS) was applied over l-M1. In one half of the trials, l-M1 TMS was preceded by a conditioning stimulus (CS) delivered 6ms earlier over a contralateral prefrontal site (r-preSMA or r-IFG), or the control site (r-M1, control experiment). This inter-stimulus interval was selected according to recent studies performed with subjects at rest reporting relevant modulation of MEP amplitude following the application of a conditioning pulse on prefrontal sites and the application of a test stimulus on M1 spaced by 6ms [9, 10]. The intensity of TS was adjusted to evoke a MEP of ~ 1 mV peak to peak in the relaxed right FDI. The CS intensity was set at the 90% of the Resting Motor Threshold (RMT) [S3]. We defined RMT as the lowest intensity that evoked five small responses (~50µV) in the contralateral FDI muscle in a series of 10 stimuli in which the subject kept the FDI muscles relaxed in both hands [S4]. The test and the conditioning stimulator were connected to a small custom-made figure-of-eight-shaped coil (external diameter, 70mm) to reduce the effective area of stimulation. The coil over M1 was always placed tangentially to the scalp at the 45° angle from midline of the central sulcus, inducing a posterior-anterior current flow. The coil position for the r-IFG and for the r-preSMA was identified and monitored during all the experiments through the SofTaxic Navigator system (Electro Medical Systems) [S4, S5]. The r-IFG and r-preSMA were determined using individual structural MRI to localize the sites and adjusted with respect to individual sulcal landmarks in each participant. In order to target r-IFG, the coil was positioned over the caudal portion of the pars opercularis of the inferior frontal gyrus, handle pointing forward. The mean Talairach coordinates used in the present study to target r-IFG were x=52.9±3.1; y=9.6±14.5; z=19.4±3.3 (Brodmann area 44). In order to target r-preSMA, the coil was positioned over the dorso-medial frontal cortex close to the paracentral sulcus approximately in line with the vertical commissure anterior and was kept with the handle pointing laterally to induce a medially directed current in the

stimulated cortex. The mean Talairach coordinates used in the present study to target r-preSMA were *x*=1± 1.4; *y*=-13±6.4; *z*=60± 4.2 (Brodmann area 6)[S6].

Main Experiment

The conditioning sites (r-IFG and r-preSMA) were tested in two different sessions spaced 1 week apart in a pseudo-randomized order. Each session consisted of four blocks of 128 trials each. In these trials, TS alone or CS+TS TMS were applied during the fixation cross presentation (pre) or at 50, 75, 100, 125, 150, 175, 200ms after the visual cue onset with a number of 8 trials collected for each Go/Nogo condition and SOA. During each block, two different SOAs were randomly selected among all possible delays (pre, 50, 75, 100, 125, 150, 175, 200ms after cue presentation). The mean peak-to-peak amplitude of the conditioned MEP was expressed as a percentage of the mean peak-to-peak amplitude size of the unconditioned TS recorded at the same SOA.

Control Experiment

A control experiment was performed in a subsample of eight subjects to ensure that the conditioning effects found in the main experiment were not due to a spread of activation from r-IFG or r-preSMA to the right M1 (r-M1). The experiment was run in a single session consisting of one block of 128 trials and two selected SOAs (50 and 75ms) with a number of 8 trials collected for each Go/Nogo condition and SOA. The CS was delivered over r-M1, whereas the TS was applied over the l-M1 as for the main experiment. The mean peak-to-peak amplitude of the conditioned MEP was then expressed as a percentage of the mean peak-to-peak amplitude size of the unconditioned TS elicited at the same SOA.

The procedure was well tolerated by all subjects. The mean 1mV threshold was 48.5±8.1% for the main experiment and 47±7.4% for the control experiment. The stimulation intensity did not differ between the two experiments (t_{13}=0.7).

TMS-EEG Experiment

Seven additional healthy volunteers (4 women and 3 men; 25±3 years old) were enrolled in a combined TMS-EEG study. This experiment was planned to further investigate whether the difference between the Go *vs.* Nogo condition could be associated to changes in beta activity driven by r-IFG or r-preSMA activation. To this aim subjects were asked to perform the Go/Nogo task as for the main experiment while the EEG signal was continuously acquired. The experiment was run in a single session consisting of one block of 128 trials (64 Go trials and 64 Nogo trials) for each conditioning site (r-IFG, r-preSMA). For each trial a single TMS pulse was applied over r-IFG or r-preSMA at 150ms from cue onset. This SOA was selected based on the main experimental results, indicating a significant difference in conditioned MEP amplitude between Go and Nogo condition at a low variability across subjects. r-IFG and r-preSMA stimulation were performed in randomized order and separated by a TMS-free interval of ~15min to allow coil replacement. TMS intensity was adjusted to 90% of RMT to match the CS intensity used in the main study.

TMS-compatible EEG equipment (BrainAmp 32MRplus, BrainProducts GmbH, Munich, Germany) was used for recording EEG activity from the scalp [7]. The EEG was continuously acquired from 32 scalp sites positioned according to the 10-20 International System, using electrodes mounted on an elastic cap. Additional electrodes were used as ground and reference. The ground electrode was positioned in AFz, while an active reference was positioned on the tip of the nose. The signal was bandpass filtered at 0.1–1000Hz and

digitized at a sampling rate of 5kHz. In order to minimize overheating of the electrodes proximal to the stimulating coil, TMS-compatible Ag/AgCl sintered ring electrodes were used. Skin/electrode impedance was maintained below 5kΩ. Horizontal EOG (HEOG) was recorded from electrodes positioned on the outer canthi of both eyes and vertical EOG (VEOG) from electrodes located beneath the right eye recorded vertical eye movements and blinks. In order to reduce auditory contamination of EEG induced by coil clicks, subjects wore earplugs throughout the experiment.

Statistical Analyses TMS Experiments

For the main experiment conditioned MEP amplitude data were analyzed by a three-way repeated measures ANOVA with Site (r-IFG *vs.* r-pre-SMA), Condition (Go *vs.* Nogo) and SOA (pre, 50, 75, 100, 125, 150, 175, 200ms) as within-subject factors.

MEPs amplitude evoked by the TS without any CS were analyzed by a two-way repeated measures ANOVA with Condition (Go *vs.* Nogo) and SOA (pre, 50, 75, 100, 125, 150, 175, 200ms) as factors. Bonferroni corrected t-tests were performed when appropriate for all experiments.

As visual inspection and statistical analysis revealed an increased l-M1 excitability peaking every 50ms in the Nogo condition (see results section), we applied a curve-fitting procedure (robust nonlinear least-squares) in a Matlab custom made code to evaluate whether a cyclic pattern could be detected [S7]. Group-averaged conditioned MEPs amplitude acquired in the Nogo trials was linearly detrended to remove linear effects across SOA and retain any cyclic patterns around the mean. We then fitted to the r-IFG or r-preSMA-conditioned data all cosine curves from 1 to 40Hz. R-squared values of the group mean data were statistically evaluated using bootstrapping. To this end, labels of the 7 SOAs (from 50 to 200ms) were

randomly permuted over 2500 iterations, and a model cosine was fitted to the resulting MEP pattern each time, generating a null distribution of 2500 R-squared values for each cosine model. The R-squared value obtained from the actual data was related to this created null-distribution to evaluate whether it fell in the top-99th percentile, indicating that the model cosine significantly explained variance in the group data. Aside from the above-described group-mean analysis, we performed a secondary analysis, in which we fitted model cosines to the behavioral data of all individual participants on the single subject-level.

Despite no difference emerged during the Go trials between any of the intervals and the pre-cue condition (rest), indicating that any change in M1 excitability was not related to the task, but likely to a general connectivity properties between the prefrontal areas and M1, we decided to apply the same fitting procedure to the data acquired in the Go trials.

For the control experiment conditioned MEPs amplitude were analyzed by repeated measures ANOVA with factors Site (r-IFG, r-preSMA, r-M1), Condition (Go *vs.* Nogo) and SOA (50 *vs.* 75ms).

Behavioral data were analyzed by two-way repeated measures ANOVA with Site (CS r-IFG, conditioned stimulation over r-IFG, CS r-preSMA, conditioned stimulation over r-preSMA, TS, test stimulation alone) and SOA (50, 75, 100, 125, 150, 175 and 200ms) as within subjects factors performed on Go RTs.

Since fixed SOAs were chosen for the application of the TMS pulses Go RTs obtained following conditioned stimulation at 50, 100 and 150ms were correlated with the normalized MEP change at the same SOAs, separately for r-IFG and r-preSMA data, to check that the significant effects obtained in MEP data are independent of the specific phase of movement preparation in which each subject was.

EEG analysis and statistics

Preprocessing. EEG signals were first re-referenced to the average of all electrodes and high-pass filtered at 0.1Hz (Butterworth zero phase filter). For each subject, condition (Go *vs.* Nogo), and conditioning area (r-IFG *vs.* r-preSMA), pre-processing epochs were of 2s duration (-1s to + 1s from TMS pulse onset). Single trials were visually inspected to exclude epochs with excessively noisy EEG, eye movement artifacts, or muscle artifacts. Because of a low quality of the EEG recording, due to exceeding artifact contaminations, one subject had to be excluded from the study before post-processing. The artifact induced by pulse delivery, typically lasting 5–6ms with our equipment [S8], was removed using cubic interpolation for a conservative 10ms interval following the TMS pulse. Independent component analysis (ICA) was then run to identify and remove components reflecting residual muscle activity, eye movements, blink-related activity, and residual TMS-related artifacts (for details about ICs exclusion criteria see [9]). No more than four components were removed in each subject.

Time-frequency analysis. For each subject, condition and site, data were averaged from 1000ms before to 1000ms after pulse onset and then submitted to a complex Morlet wavelet (2–40Hz, 20 frequency steps, c=7). The frequency layers which significantly fitted (see fitting results) the MEPs modulation were then extracted from the wavelet dataset (central frequencies: 18.1 to 23.03Hz) for the electrodes closest to l-M1 (C3) and r-IPF (FC2) and r-SMA (FCz). The evoked activity over C3 was analyzed by means of repeated measure ANOVA with factors Condition (Go *vs.* Nogo) and Site of TMS (r-IFG *vs.* r-preSMA).

Source localization. Frequency analysis was performed for all clean trials collected during the r-IFG stimulation across all participants. Single trial data from a 250ms long window centered at latency 125ms was weighted with a Hanning window and subjected to FFT. The

cross spectral density matrix for all sensor combinations was computed for beta frequency at 20Hz both, for the Go and Nogo condition. Finally, the standard FieldTrip EEG volume conductor model was used together with the DICS beamformer on an 8mm grid to compute the spatial distribution of beta power for both conditions [S9].

Phase Locking Values. To investigate functional connectivity between r-IFG and l-M1 the phase locking value over time across all clean trials [S10] was computed. DICS beamformer coefficients were recomputed for a longer time window for the two target areas (for coordinates see Paired Pulse Stimulation paradigm above). Single trial data were bandpass filtered in the beta band and multiplied with beamformer coefficients to obtain time series of beta band activity for both regions of interest. Instantaneous phase was then computed via the Hilbert transform and the phase locking value was calculated for Nogo and Go condition by using the standard formula [S10]. The 99% confidence level for phase locking was computed by random permutation of single trials. The randomization was performed 1000 times. Finally, phase locking time series was plotted in units of standard deviation of the baseline [S11].

Phase Locking Index (PLI). To test if the cyclic pattern found for the MEP modulation in the Nogo condition could be explained by a local increase in beta phase consistency across trials, we computed PLI on artifact-free data for all frequencies from 2 to 30 Hz for the electrode closest to the left motor cortex (C3) (for single pulse TMS over the right IFG). To this end, data were submitted to a time frequency analysis (Hanning windowed) and PLI was then calculated for the Nogo and the Go condition separately by using the standard formula [S12]. The PLI values extracted for each condition were compared by means of a paired t-test.

Supplemental Results

MEP-measure of effective connectivity: single-subject data in Nogo trials

To verify whether the beta band models, which significantly fitted the group-averaged conditioned MEPs collected during the Nogo trials, also apply to individual data, we repeated the fitting procedure for each participant. When the CS was applied over the r-IFG, MEP amplitude modulations were significantly explained by a cosine model cycling at beta frequency for 9 (out of 10) participants (Figure S1A). More specifically, the same beta cosine models used for the group data (range 17-23Hz) also fitted the individual data of S1, S3-S4, S6-S8 and S10, while beta model ranges had to be slightly adapted for S2 (15-21Hz) and S5 (16-24Hz) to obtain significant fitting (bootstrapped 99%-cut-offs). No cosine model significantly explained MEPs data collected from S9.

When the CS was applied over the r-preSMA, single subject analysis revealed that the original cosine models in the beta-frequency range (from 17-23Hz) significantly fitted the MEP data of 8 (out of 10) participants (bootstrapped 99%-cut-off; Figure S1B). No significant fitting was obtained for S7 and S9.

MEP-measure of effective connectivity: group-averaged and single-subject data in Go trials

An additional fitting procedure was performed on group averaged conditioned MEPs data collected during the Go trial. Permutation tests revealed that cosine models in the beta-frequency range (from 19-21Hz) significantly fitted the MEP-data when the CS was delivered over r-preSMA (bootstrapped 99%-cut-off, 20Hz cosine being the model explaining the highest percentage of variance that is 66%; Figure S2A). No significant result emerged for

the r-IFG condition. However, when single subject data were subjected to the same fitting procedure, only data from 3 participants were significantly explained by the beta band cosine model (Figure S2B).

Performance measures (accuracy and RT) over SOAs in Go trials

In all conditions behavioural performance was highly accurate with low variability (accuracy Go trials: 99.3±1.1%; false alarms Nogo trials: 2.5±2.1%), also across conditions. For this reason both accuracy scores and false alarms RTs were not statistically analysed.

Analysis of reaction times (RT, two-way ANOVA on Go-trials only) showed a significant main effect of SOA ($F_{6,54}$=6.90; p<0.001). No other main effect or interaction reached significance. *Post-hoc* comparisons indicated that Go RTs were slowed down at 100 (all p <0.001) and 150 (all p<0.005) ms after the stimulus presentation with respect to the other SOAs, regardless of conditioning (Figure S3).

Performance measures: RT *vs.* MEP changes in Go trials

To probe whether the significant effects obtained in MEP data are independent of the specific phase of movement preparation in which each subject was, Go RTs obtained following paired-pulse stimulation at 50, 100 and 150ms (every second SOA) were correlated with the normalized MEP change at the same SOAs, separately for r-IFG and r-preSMA data. No significant correlation emerged (r-IFG SOA 50: r= -0.145; r-preSMA SOA 50: r=0.128; r-IFG SOA 100: r=0.274; r-preSMA SOA 100: r=-0.171; r-IFG SOA 150: r=-0.077; r-preSMA SOA 150 r=0.417; all p> 0.05).

Phase-locking index: Nogo vs Go condition

To test whether the oscillatory pattern found for the MEP amplitude cycling at beta frequency could reflect an increased inter-trial beta phase consistency across the Nogo- as

compared to the Go-trials, the PLI was calculated for both conditions and compared by means of a paired t-test. Figure S4 shows the difference between the two conditions (Nogo minus Go) when the TMS was applied over the r-IFG. Our results show that PLI was selectively increased around 20Hz in the Nogo trials. This increase was significant ($t(6)= 3.28$; $p=0.016$).

Supplemental References

S1. Oldfield, R.C. (1971). The assessment and analysis of handedness: the Edinburgh inventory. Neuropsychologia *9*, 97-113.

S2. Pandey, A.K., Kamarajan, C., Tang, Y., Chorlian, D.B., Roopesh, B.N., Manz, N., Stimus, A., Rangaswamy, M., and Porjesz, B. (2012). Neurocognitive deficits in male alcoholics: an ERP/sLORETA analysis of the N2 component in an equal probability Go/NoGo task. Biol Psychol. *89*, 170-182.

S3. Rossini, P.M., Barker, A.T., Berardelli, A., Caramia, M.D., Caruso, G., Cracco, R.Q., Dimitrijevic, M.R., Hallett, M., Katayama, Y., Lucking, C.H., et al. (1994). Non-invasive electrical and magnetic stimulation of the brain, spinal cord and roots: basic principles and procedures for routine clinical application. Report of an IFCN committee. Electroencephalogr Clin Neurophysiol. *91*, 79-92.

S4. Koch, G., Ponzo, V., Di Lorenzo, F., Caltagirone, C., and Veniero, D. (2013). Hebbian and anti-Hebbian spike-timing-dependent plasticity of human cortico-cortical connections. J Neurosci. *33*, 9725-9733.

S5. Koch, G., Cercignani, M., Pecchioli, C., Versace, V., Oliveri, M., Caltagirone, C., Rothwell, J., and Bozzali, M. (2010). In vivo definition of parieto-motor connections involved in planning of grasping movements. NeuroImage *51*, 300-312.

S6. Nachev, P., Kennard, C., and Husain, M. (2008). Functional role of the supplementary and pre-supplementary motor areas. Nat Rev Neurosci. *9*, 856-869.

S7. de Graaf, T.A., Gross, J., Paterson, G., Rusch, T., Sack, A.T., and Thut, G. (2013). Alpha-band rhythms in visual task performance: phase-locking by rhythmic sensory stimulation. PLoS One *8*, e60035.

S8. Veniero, D., Bortoletto, M., and Miniussi, C. (2009). TMS-EEG co-registration: on TMS-induced artifact. Clin Neurophysiol. *120*, 1392-1399.

S9. Gross, J., Kujala, J., Hamalainen, M., Timmermann, L., Schnitzler, A., and Salmelin, R. (2001). Dynamic imaging of coherent sources: Studying neural interactions in the human brain. Proc Natl Acad Sci U S A. 98, 694-9.

S10. Lachaux, J.P., Rodriguez, E., Martinerie, J., and Varela, F.J. (1999). Measuring phase synchrony in brain signals. Hum Brain Mapp. 8, 194-208.

S11. Rodriguez, E., George, N., Lachaux, J.P., Martinerie, J., Renault, B., and Varela, F.J. (1999). Perception's shadow: long-distance synchronization of human brain activity. Nature *397,* 430-3.

S12. Tallon-Baudry, C., Bertrand, O., Delpuech, C., Pernier, J. (1996). Stimulus Specificity of Phase-Locked and Non-Phase-Locked 40 Hz Visual Responses in Human. J Neurosci. *16*, 4240-4249.
